# The trypanocidal benzoxaborole AN7973 inhibits trypanosome mRNA processing

**DOI:** 10.1371/journal.ppat.1007315

**Published:** 2018-09-25

**Authors:** Daniela Begolo, Isabel M. Vincent, Federica Giordani, Ina Pöhner, Michael J. Witty, Timothy G. Rowan, Zakaria Bengaly, Kirsten Gillingwater, Yvonne Freund, Rebecca C. Wade, Michael P. Barrett, Christine Clayton

**Affiliations:** 1 Center for Molecular Biology of Heidelberg University (ZMBH), DKFZ-ZMBH Alliance, Im Neuenheimer Feld 282, Heidelberg, Germany; 2 Wellcome Centre for Molecular Parasitology, Institute of Infection, Immunity and Inflammation, College of Medical, Veterinary and Life Sciences, 120 University Place, University of Glasgow, Glasgow, United Kingdom; 3 Molecular and Cellular Modeling Group, Heidelberg Institute for Theoretical Studies (HITS), Schloß-Wolfsbrunnenweg 35, Heidelberg, Germany; 4 Global Alliance for Livestock and Veterinary Medicine, Doherty Building, Pentlands Science Park, Penicuik, Edinburgh, United Kingdom; 5 Centre International de Recherche–Développement sur l’Elevage en zone Subhumide (CIRDES), Bobo-Dioulasso 01, Burkina Faso; 6 Swiss Tropical and Public Health Institute, Socinstrasse 57, Basel, Switzerland; 7 University of Basel, Petersplatz 1, Basel, Switzerland; 8 Anacor Pharmaceuticals, Inc., Palo Alto, CA, United States of America; 9 Interdisciplinary Center for Scientific Computing (IWR), Heidelberg University, Im Neuenheimer Feld 205, Heidelberg, Germany; 10 Glasgow Polyomics, University of Glasgow, Glasgow, United Kingdom; University of Texas Southwestern Medical School, UNITED STATES

## Abstract

Kinetoplastid parasites—trypanosomes and leishmanias—infect millions of humans and cause economically devastating diseases of livestock, and the few existing drugs have serious deficiencies. Benzoxaborole-based compounds are very promising potential novel anti-trypanosomal therapies, with candidates already in human and animal clinical trials. We investigated the mechanism of action of several benzoxaboroles, including AN7973, an early candidate for veterinary trypanosomosis. In all kinetoplastids, transcription is polycistronic. Individual mRNA 5'-ends are created by *trans* splicing of a short leader sequence, with coupled polyadenylation of the preceding mRNA. Treatment of *Trypanosoma brucei* with AN7973 inhibited *trans* splicing within 1h, as judged by loss of the Y-structure splicing intermediate, reduced levels of mRNA, and accumulation of peri-nuclear granules. Methylation of the spliced leader precursor RNA was not affected, but more prolonged AN7973 treatment caused an increase in S-adenosyl methionine and methylated lysine. Together, the results indicate that mRNA processing is a primary target of AN7973. Polyadenylation is required for kinetoplastid *trans* splicing, and the EC_50_ for AN7973 in *T*. *brucei* was increased three-fold by over-expression of the *T*. *brucei* cleavage and polyadenylation factor CPSF3, identifying CPSF3 as a potential molecular target. Molecular modeling results suggested that inhibition of CPSF3 by AN7973 is feasible. Our results thus chemically validate mRNA processing as a viable drug target in trypanosomes. Several other benzoxaboroles showed metabolomic and splicing effects that were similar to those of AN7973, identifying splicing inhibition as a common mode of action and suggesting that it might be linked to subsequent changes in methylated metabolites. Granule formation, splicing inhibition and resistance after CPSF3 expression did not, however, always correlate and prolonged selection of trypanosomes in AN7973 resulted in only 1.5-fold resistance. It is therefore possible that the modes of action of oxaboroles that target trypanosome mRNA processing might extend beyond CPSF3 inhibition.

## Author summary

Trypanosomes and leishmanias infect millions of humans and cause economically devastating diseases of livestock; the few existing drugs have serious deficiencies. Trypanosomosis of cattle caused by *Trypanosoma congolense* and *Trypanosoma vivax*, is a serious problem in Africa because cattle are used not only for food but also for traction, and new drugs are needed. A single injection of the benzoxaborole compound AN7973 cured *T*. *congolense* infection in cattle and goats. Although slightly lower effectiveness against *T*. *vivax* precludes development of AN7973 as a commercially viable treatment against cattle trypanosomosis, it could still have potential for diseases caused by other trypanosomes. We used a large range of methods to find out how AN7973 kills trypanosomes, and compared it with other benzoxaboroles. AN7973 and some of the other compounds had effects on parasite metabolism that resembled those previously seen for a benzoxaborole that is being tested for human sleeping sickness. The most rapid effect of AN7973, however, was on processing of trypanosome mRNA. As a consequence, amounts of mRNA decreased and synthesis of proteins stopped. We conclude that AN7973 and some other benzoxaboroles kill trypanosomes by stopping gene expression.

## Introduction

Kinetoplastid protists cause severe human diseases affecting millions of people. *Trypanosoma cruzi* causes Chagas disease in South America, and various *Leishmania* species cause a spectrum of diseases throughout the tropics. Salivarian trypanosomes, the subject of this study, cause sleeping sickness in humans and economically important diseases in cattle, horses and camels [1–3]. Approximately 70 million people, living in sub- Saharan Africa, are estimated to be at risk of contracting human African trypanosomosis, which is caused by *Trypanosoma brucei* subspecies [4, 5]. As a result of sustained international activities to control the disease [4–6], less than 3000 cases were reported in 2016 (http://www.who.int/trypanosomiasis_african/en/). Trypanosomosis in cattle, caused by infection with *Trypanosoma congolense*, *Trypanosoma vivax* and, to a lesser extent, *T*. *brucei*, is in contrast a major problem, with wide-reaching effects on human well-being: cattle are used not only as a source of milk and meat, but also for traction. Elimination of cattle trypanosomosis could create economic benefits estimated at nearly 2.5 billion US$ per year [2]. Within Africa, trypanosomosis is transmitted by tsetse flies, but outside Africa, variants of *T*. *brucei* are transmitted venereally or by biting flies, and *T*. *vivax* can also be transmitted non cyclically by non-tsetse biting flies with massive economic losses affecting draught and milk animals from Argentina to the Philippines [7].

Control of cattle trypanosomosis currently relies on reducing the tsetse population by means of traps and insecticidal dips, together with treatment as required. The most popular treatment is with the diamidine diminazene aceturate (Berenil), the alternative being the DNA-intercalating molecule isometamidium [3]. Suramin is also sometimes used [3]. Development of new animal therapeutics is constrained by the need for cure after a single intramuscular injection [3].

In the last ten years, benzoxaboroles have generated considerable excitement for antimicrobial and other applications. Benzoxaboroles have a range of known effects. For example, they are able to bind *cis*-diols, such as those found in sugars, yielding stable spiro complexes [8]. This activity is the basis of the mode of action of the antifungal drug Tavaborole (AN2690) [9], which binds to the editing site of leucyl tRNA synthetase [10]. Other oxaboroles inhibit bacterial leucyl tRNA synthetases by the same mechanism [11–13]. Other benzoxaborole classes interact with ATP-binding pockets, but with different modes of binding. Crisaborole, approved for the treatment of atopic dermatitis [14, 15], selectively inhibits phosphodiesterase PDE4, with the oxaborale oxygen atoms coordinating the zinc and magnesium ions within the active site [16]. The aminomethylphenoxy benzoxaboroles inhibit Rho-activated protein kinases through hydrogen bond interactions with the hinge region of the protein and the aminomethyl group interacting with the magnesium/ATP-interacting aspartic acid [17].

**Fig 1:**
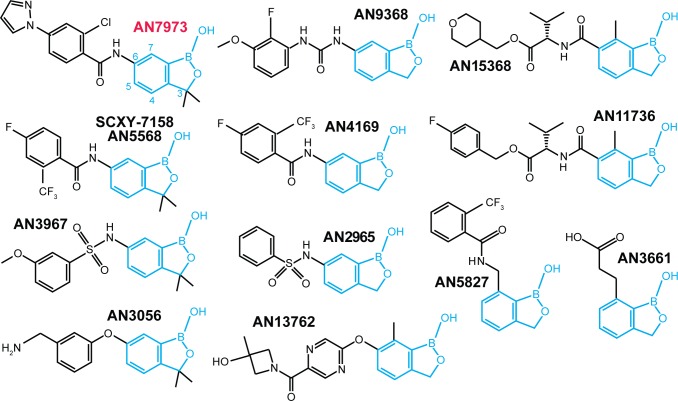
Structures of benzoxaboroles studied in this paper. The benzoxaborole scaffold is in cyan.

The first trypanocidal oxaboroles to be described were the oxaborole 6-carboxamides [18, 19]. Acoziborole (trade name of SCYX-7158 or AN5568, 1) [20] is orally available and can cross the blood brain barrier [18]; it is now in phase IIb/III clinical trials for sleeping sickness (see https://www.dndi.org/diseases-projects/portfolio/). AN7973 (Fig 1), the main subject of this paper, is efficacious against *T*. *congolense* and *T*. *brucei* and was considered as a candidate for treatment of cattle trypanosomosis, but was later replaced by AN11736 (Fig 1), which can achieve single-dose cure of both *T*. *congolense* and *T*. *vivax* infection in cattle [21].

At present, not enough is known about structure-activity relationships in benzoxaboroles to enable predictions to be made about their modes of action. Jones et al. [22] obtained *T*. *brucei* lines with 4-5-fold resistance to AN2965 (Fig 1, named oxaborole-1 in their paper). They observed numerous single nucleotide polymorphisms (SNPs), genome rearrangements and amplifications in the resistant lines. Affinity purification with a benzoxaborole column yielded 14 proteins that bound specifically, but since none of these was affected in the resistant mutants, there was no clear indication as to which might be relevant to benzoxaborole action [22]. Aminomethyl phenoxyl benzoxaboroles such as AN3056 are sequentially activated to an active carboxylic acid form by serum and intracellular enzymes [23].

Various oxaboroles are being considered for treatment of apicomplexan parasites; and AN13762 (Fig 1) is in development [24, 25]. *Plasmodium* and *Toxoplasma* that were resistant to AN3661 (Fig 1) had mutations in the cleavage and polyadenylation factor CPSF3 [26, 27], which is implicated in 3' cleavage of mRNA precursors prior to polyadenylation [28]. The two zinc ions of CPSF3 are thought to interact with phosphate [28]. *In silico* molecular docking of AN3661 suggested that the boron atom occupies the position of the cleavage site phosphate of the mRNA substrate, with one hydroxyl group interacting with a zinc atom in the catalytic site—similar to binding of other benzoxaboroles to the bimetal centers of beta-lactamase and phosphodiesterase-4 [26, 27]. Introduction of resistance mutations—which were all in or near the active site—into susceptible parasites resulted in compound resistance. These results, combined with the loss of transcripts for three trophozolite-expressed genes in treated parasites, suggest that AN3661 inhibits mRNA polyadenylation through its interaction with CPSF3 [26, 27].

In trypanosomes, transcription of mRNAs is polycistronic. Individual mRNA 5'-ends are created co-transcriptionally by *trans* splicing of a 39nt leader sequence (*SL*) [29–31]. *Trans* splicing is spatially and mechanistically coupled to polyadenylation of the preceding mRNA. Polyadenylation sites are dictated by the positions of *trans*-splicing sites [32–35], RNAi-mediated depletion of polyadenylation factors inhibits *trans* splicing [36, 37], and disruption of splicing stops polyadenylation [32–35]. The spliced leader precursor RNA (*SLRNA*) is 139 nt long and is synthesised by RNA polymerase II [38] from approximately 200 tandemly repeated genes [39]. Unlike protein-coding genes, each *SLRNA* gene has its own promoter [40–42]. The *SLRNA* cap and the following four nucleotides are methylated [43–46]. Inhibition of *SLRNA* methylation using S-adenosyl-L-homocysteine or Sinefungin prevents splicing [47, 48]. This results in loss of mRNA from the cells by the normal mechanisms of mRNA turnover [49]. It has recently been shown that an accumulation of numerous metabolites, including S-adenosylmethionine, occurs in trypanosomes treated with Sinefungin, and a similar profile was identified in trypanosomes treated with acoziborole [50].

We here describe studies to discover molecular targets of several anti-trypanosomal benzoxaboroles. Our work concentrated on AN7973 (Fig 1), which is orally active and was the DNDi back-up for SCYX-7158/AN5568 for the treatment of human African trypanosomosis. We undertook a comprehensive analysis, including resistance generation and characterisation of morphology, metabolomes, macromolecular biosynthesis and molecular modeling.

## Results

### Anti-trypanosomal activities of AN7973

AN7973 (Fig 1) was selected as a candidate veterinary drug from a range of 7-carboxamido-benzoxaboroles structurally similar to AN5568. The selection of AN7973 was based on its *in vitro* potency against *T*. *congolense* (S1 Table, sheet 1) and its ability to cure *T*. *congolense*-infected mice with a single 10mg/kg i.p. dose (S1 Table, sheet 3).*T*. *congolense*-infected goats were also cured when AN7973 was administered as a single bolus dose injection of 10 mg/kg, but for *T*. *vivax*-infected goats, two intramuscular injections of 10 mg/kg were required (S1 Table, sheet 3). Testing in cattle was done using *T*. *vivax* and *T*. *congolense* isolates that were resistant to maximum dosages of diminazene (7 mg/kg) and isometamidium (1 mg/kg). A single 10 mg/kg intramuscular injection of AN7973 cured 3/3 cattle of *T*. *congolense* infection, but two 10 mg/kg injections of AN7973 cured only one out of two *T*. *vivax* infections and a single injection failed to cure 3 animals (S1 Table, sheet 3). This meant that AN7973 would be inappropriate for field use [3]. The reduced efficacy of AN7973 against *T*. *vivax* might have been a consequence of weaker intrinsic potency: the *ex vivo* EC_50_ against *T*. *vivax* was 215 nM, as against an *in vitro* EC_50_ of 84 nM for *T*. *congolense* (S1 Table, sheet 1). The latter value is similar to the results for *T*. *brucei* (20–80 nM, S1 Table).

The lack of single-dose cattle efficacy at 10mg/kg i.m. against *T*. *vivax* precluded development of AN7973 as a commercially viable treatment against cattle trypanosomosis, but it could still have potential for diseases caused by other salivarian trypanosomes.

### Resistance selection, cell cycle and protein synthesis

One of the most direct strategies to identify the targets of antimicrobial drugs is selection of resistant mutants. We therefore attempted to select parasites resistant to AN7973 using an over-expression library [51]. The level of resistance obtained was very modest—less than 2-fold (S2 Table, sheet 1) and in the two lines that we obtained, the over-expression plasmids did not contain an in-frame coding sequence. We therefore sequenced their genomic DNA. As previously observed for trypanosomes that were mildly resistant to AN2965 [22] we found numerous single nucleotide polymorphisms, amplifications and deletions (summarized in S2 Table, details in S3 and S4 Tables). The large number of genes affected made it impossible to pinpoint any particular pathway as being relevant to resistance.

**Fig 2:**
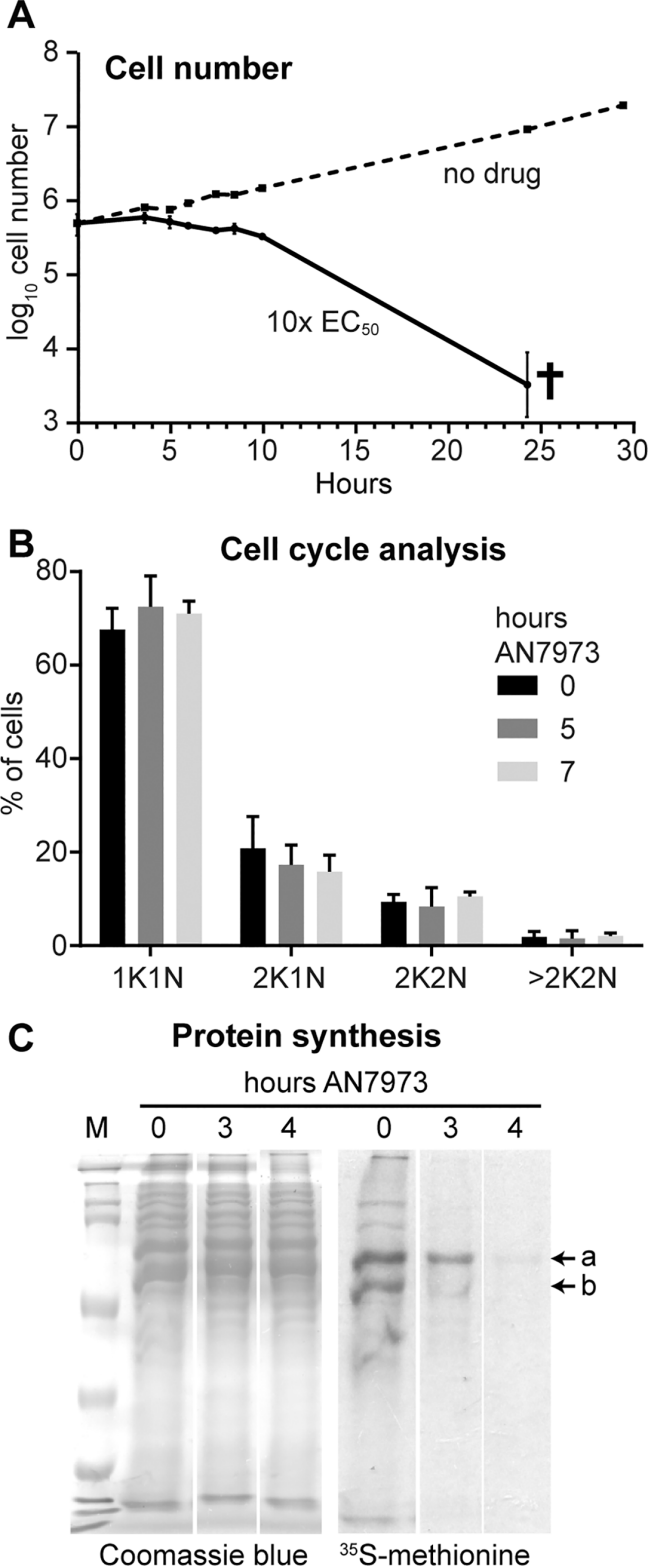
Effects of AN7973 on the cell cycle and protein synthesis. A) Cumulative growth curve for bloodstream-form trypanosomes incubated with and without AN7973. Results are arithmetic mean ± standard deviation for three replicates. B) Cell cycle analysis by microscopy. The percentages of cells with different numbers of nuclei and kinetoplasts are shown. C) Effect on protein synthesis. 5x10^6^ Cells were incubated with AN7973 (10x EC_50_) for various times, then (after washing and 15-min pre-incubation) with [^35^S]-methionine for 30 min. Proteins were separated by SDS-PAGE and radioactive incorporation was assessed by autoradiography. The Coomassie-stained gel is shown as a loading control. Two prominent protein bands showing different effects of AN7973 are indicated as "a" and "b". All lanes are from the same gel and exposure; the gaps between the lanes are present because identical results for two partially resistant lines (also treated at 10x EC_50_) have been deleted.

Examination of parasite morphology can reveal defects in DNA synthesis, cell cycle regulation, cell motility and protein trafficking, each of which can ultimately cause parasite death. For example, Jones et al. saw accumulation of parasites in the G2 phase of the cell cycle after AN2965 treatment [22]. Treatment of trypanosomes with AN7973 with 10x EC_50_ for 7h caused growth arrest (Fig 2A), but no obvious changes in parasite morphology or motility and no significant changes in the proportions of cells in different stages of the cell cycle (Fig 2B). These results suggest that AN7973 does not interfere directly with DNA synthesis, cell motility, or protein trafficking.

Amino-acyl tRNA synthetase inhibition is a known mode of action of several benzoxaboroles, and inhibition of tRNA synthetase should result in cessation of protein synthesis. We therefore measured this in AN7973-treated trypanosomes by pulse labelling with [^35^S]-methionine followed by denaturing polyacrylamide gel electrophoresis and autoradiography. Inhibition of protein synthesis (Fig 2C) was clear, but some prominent protein bands were affected more than others (compare bands labeled a and b). The kinetics of the inhibition, combined with apparent selectivity for particular proteins, suggested that protein synthesis was not a primary target of AN7973, but might be inhibited in a secondary fashion.

### AN7973 inhibits mRNA processing

**Fig 3:**
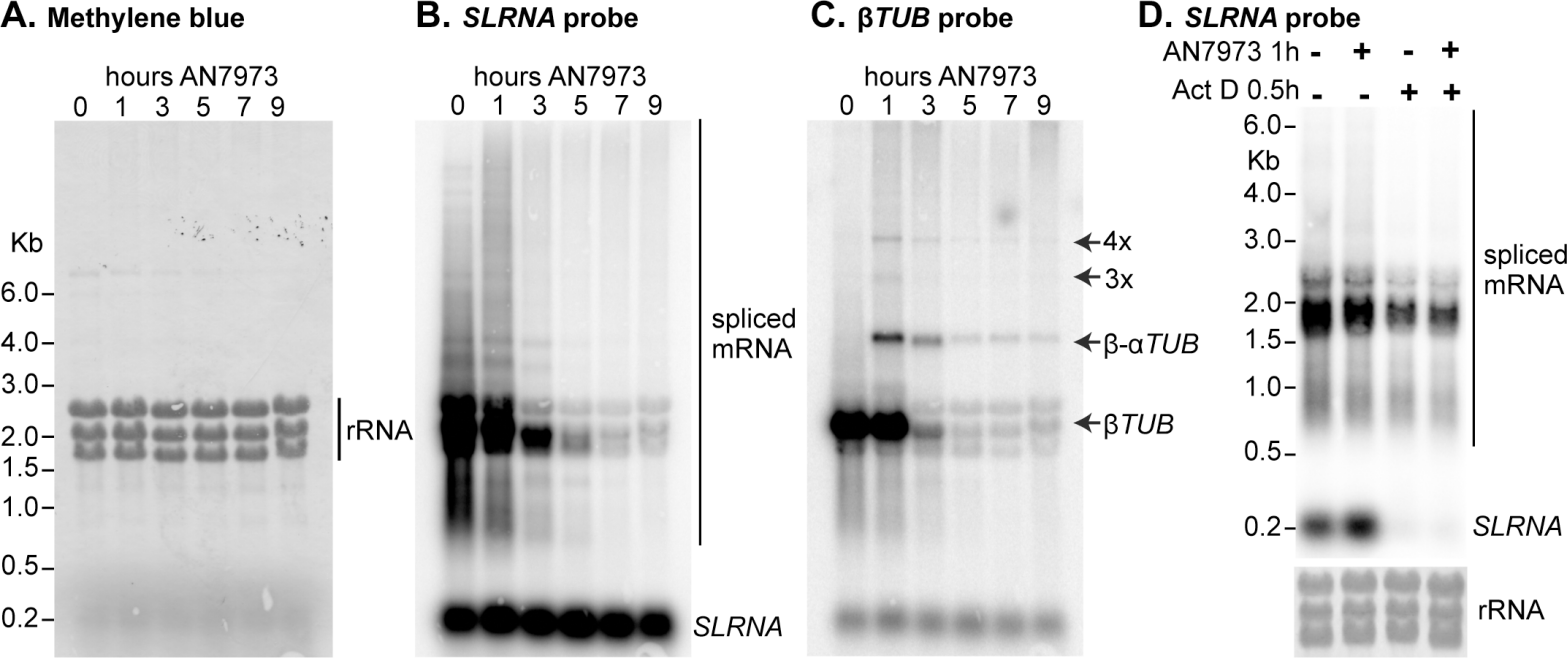
AN7973 affects mRNA processing. A) Trypanosomes were incubated with AN7973 for different times (0–9 hours). RNA was subjected to denaturing agarose gel electrophoresis, blotted, and detected using radioactive probes. This panel shows Methylene blue-stained gel showing equal loading of total RNA. The three prominent bands are rRNA. B) As (A), blot probed with a [^32^P]-end-labelled probe complementary to the spliced leader. The signals representing the ~140nt *SLRNA* and mature mRNAs are indicated. The cluster of bands around 2 kb probably represent the abundant mRNAs encoding the Variant Surface Glycoprotein, alpha and beta tubulin (both ~1.9 kb including 100nt poly(A)), and EF-1 alpha (1.8 kb) [91]. C) The blot from (B) was stripped then incubated with a beta-tubulin probe. Shadows from the *SL* probe remain. The positions of monomer, dimer and multimers from the tubulin repeats are indicated. D) The effect of AN7973 is not due to inhibition of *SLRNA* synthesis. Trypanosomes were treated either with AN7973 for 1h, or with Actinomycin D for 30 min, or with AN7973 followed by Actinomycin D, as indicated above the lanes. The lower panel is the rRNA loading control.

Loss of protein synthesis could be caused by loss of mRNA. For example, if mRNA synthesis were inhibited, the pattern in Fig 2C would be explained if the mRNA encoding protein "b" were less stable than that encoding protein "a". Loss of mRNA could be caused by inhibition of either RNA transcription or processing. To investigate this possible mechanism, we incubated cells with AN7973, prepared RNA, and hybridised Northern blots with a probe that detects the spliced leader *SL*. This probe detects all processed mRNAs, as well as the ~139nt precursor, called *SLRNA*, that donates the *SL*. Incubation with AN7973 for 9h had little or no effect on the total amount of rRNA, as judged by methylene blue staining (Fig 3A) but caused progressive loss of spliced mRNA (Fig 3B). In contrast, the levels of the *SLRNA* remained roughly constant (Fig 3B). We therefore suspected that AN7973 was inhibiting mRNA processing. To test this we re-hybridised the blot with a probe that detects beta-tubulin. The tubulin genes are arranged in alternating alpha-beta tandem repeats, and splicing inhibition through heat shock [52] or Sinefungin treatment [47] leads to accumulation of partially processed beta-alpha dimers and multimers. Partially processed tubulin mRNAs were indeed detected within an hour of AN7973 application (Fig 3C). This result showed that after AN7973 addition, transcription of protein-coding genes continued but mRNA processing was impaired. At later time points, the tubulin mRNAs disappeared, suggesting that the failure in processing was complete. (Most measurements suggest that the tubulin mRNAs have half-lives of about half an hour [53].)

As noted above, after 1h AN7973 treatment, the level of *SLRNA* was not much changed. This is somewhat counter-intuitive, since one might expect processing inhibition to lead to a build-up of *SLRNA*. However, RNAi that targets splicing or polyadenylation factors rarely causes *SLRNA* increases of more than 2-fold [37, 54, 55]. The reason is that *SLRNA* synthesis is balanced by degradation. Thus when Actinomycin D (Act D) is used to inhibit transcription, *SLRNA* disappears within 30 min even though no new precursors are available for splicing [56]. Fig 3D compares the effects of AN7973 with those of Act D treatment. As expected, 30 min Act D resulted in *SLRNA* loss whereas AN7973 had no effect. Moreover, if Act D was added after a one-hour AN7973 treatment, *SLRNA* again disappeared within 30 min—whereas *SLRNA* was still visible after 9h in the presence of AN7973. We concluded that treatment of the cells with AN7973 for only one hour inhibited mRNA processing but did not prevent synthesis of *SLRNA*.

Inhibition of procyclic trypanosome mRNA processing by RNAi targeting polyadenylation factors causes growth arrest within 3 days, followed by cell death [37, 54]. These delays in observing effects are explained by the fact that the targeted proteins remain detectable (see e.g. [55]). Processing inhibition can therefore easily explain killing of trypanosomes by AN7973.

### mRNA processing inhibition by AN7973 is not due to loss of cap methylation

**Fig 4:**
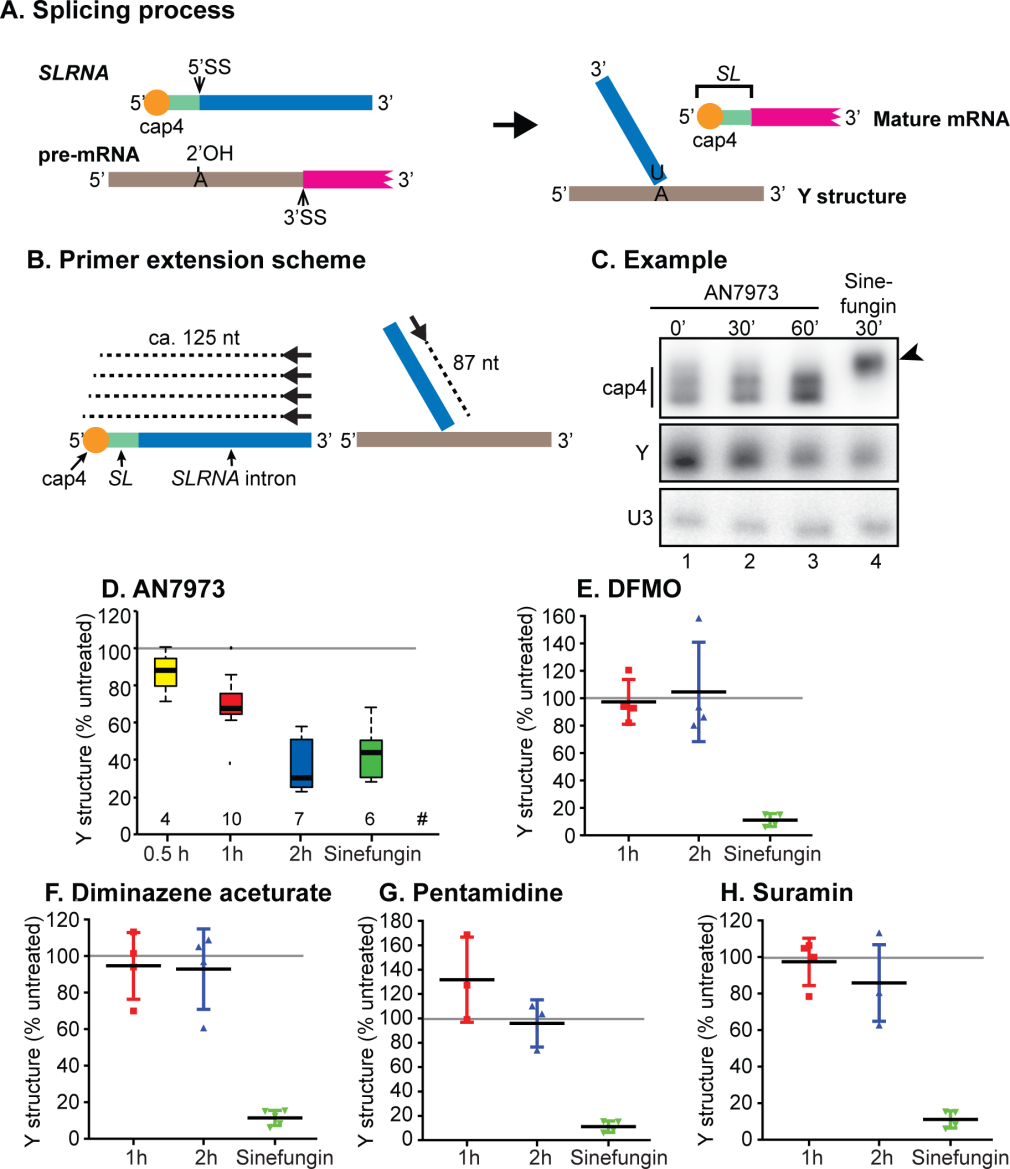
Effect of AN7973 on *trans*-splicing. A) Schematic representation of the splicing reaction. *SLRNA* and Y structure during primer extension experiments. *SLRNA* is represented with various colours; red is the cap4 region, green is the spliced leader (*SL*), blue is the *SL* intron. The pre-mRNA is shown with a grey intergenic region and a magenta portion that represents the 5' end of a mature mRNA. The 5' and 3' ends of each RNA are indicated. During *trans* splicing, the SL intron forms a branched "Y" structure with the 2' hydroxyl of an adenosine located 5' to the polypyrimidine tract that is recognised by the splicing machinery. Meanwhile the *SL* is *trans* spliced to the 5'-end of the mRNA. B) Schematic representation of the primer extension assay. The 5'-end labelled oligonucleotide primer hybridises towards the 3' end of the *SLRNA*. Reverse transcription on the intact *SLRNA* extends (dashed line) towards the 5' end but terminates upon encountering the methylated residues of cap4. In the Y structure, the primer extends (dashed line) until the branch point, giving an 87nt product. C) Typical result from a primer extension experiment illustrated in (B). A primer complementary to the U3 snRNA was used as a control. Lane 1 (0’) shows the result from trypanosomes that were treated with DMSO only. Lanes 2 and 3 show the results from trypanosomes treated with AN7973 for 30 min and 60 min, and lane 4 is the result for 30 min Sinefungin. The arrowhead shows the primer extension product from unmethylated *SLRNA*. D) Quantification from several independent tests of AN7973 treatment; the ratio between Y structure and the *U3* signal is shown. # indicates the number of data points used. Sinefungin results were taken only from experiments that included AN7973 on the same gel. The central line is the median, and boxes extend from the 25th to the 75th quartile. The whiskers extend to the most extreme data point that is no more than 1.5 times the inter-quartile range. Other points are outliers. Details for our initial experiments are in S1A and S1B Fig. After only 30 min incubation with AN7973, the difference between treated and untreated cells was significant using a one-way ANOVA. E-H) Trypanosomes were incubated with 10x EC50 of various known anti-trypanosomal drugs for 1h or 2h, then splicing was assayed. The % Y structure (relative to U3) is shown (individual points, plus mean and standard deviation). The 95% confidence intervals are shown in S1C–S1F Fig.

The only small molecule currently known to inhibit kinetoplastid mRNA processing *in vivo* is the S-adenosyl methionine analogue Sinefungin, which is a general methylation inhibitor [57]. Chemical inhibition of spliced leader methylation prevents *trans* splicing in a permeabilised cell system [48], and Sinefungin does the same *in vivo* [47]. Since we already knew that AN5568 caused increases in methylated intermediates [50], we wondered whether splicing inhibition by AN7973 was also caused by inhibition of spliced leader methylation. We used primer extension to assess the amount and methylation status of *SLRNA*. At the same time, we assayed the level of the 2'-5' branched "Y-structure" splicing product (Fig 4A). cDNA synthesis was primed by a 5'-labelled oligonucleotide that is complementary to a region towards the 3' end of the *SLRNA* (black arrows in Fig 4B). The products from full-length *SLRNA* form a small ladder, because 5' methylation partially blocks reverse transcriptase, while the Y-structure *trans* splicing intermediate gives a product of 87nt (Fig 4B). These products are seen in Fig 4C, lane 1. As expected, incubation with the methylation inhibitor Sinefungin for 30 min abolished the multiple bands caused by cap methylation, with cDNA synthesis extending cleanly to the 5' end of the *SLRNA* (Fig 4C, lane 4, arrowhead); at the same time, the signal from the Y structure was decreased. The concentration of Sinefungin used, 2 μg/mL, is 4000 times the EC_50_. This is the standard concentration in these types of experiments and was chosen in order to prevent cap methylation within about 10 min [58].

Importantly, AN7973 (10x EC_50_) had no effect on the pattern of bands from the *SLRNA*, showing that cap methylation was not affected (Fig 4C). In contrast, Y-structure formation was clearly decreased (Fig 4C, lanes 2 and 3). The combined results so far thus showed that AN7973 acts specifically on splicing, not on production of mature splicing-competent *SLRNA*. Quantitation of four independent experiments showed that after 30 min, the effect of AN7973 was already significant (p<0.0005, S3A Fig). Remarkably, considering the differences in the doses used, a 2-h incubation with AN7973 inhibited Y-structure formation to the same extent as a 30-min incubation with Sinefungin (Fig 4D).

Although the effect of AN7973 on splicing was quite rapid, we nevertheless had to consider the possibility that it was secondary to inhibition of some other process. Apart from Sinefungin, the only treatment that was previously shown to cause accumulation of tubulin precursors within 30 min was severe heat shock [52, 59], but the mechanism is unknown and there are numerous other effects including general translation arrest [59] and transcription inhibition [60]. To find out whether *trans* splicing inhibition was a general feature of parasites stressed through addition of trypanocidal or trypanostatic drugs, we measured Y structure abundance in cells treated with DFMO, diminazene aceturate, pentamidine or suramin, all at 10x EC_50_. No significant inhibition of Y-structure formation was observed (Fig 4E–4H, S3 Fig). This result demonstrated conclusively that splicing inhibition is not a general side-effect of growth inhibition. The effect of AN7973 on splicing therefore indicates a specific mechanism.

### Metabolic effects of AN7973

**Fig 5:**
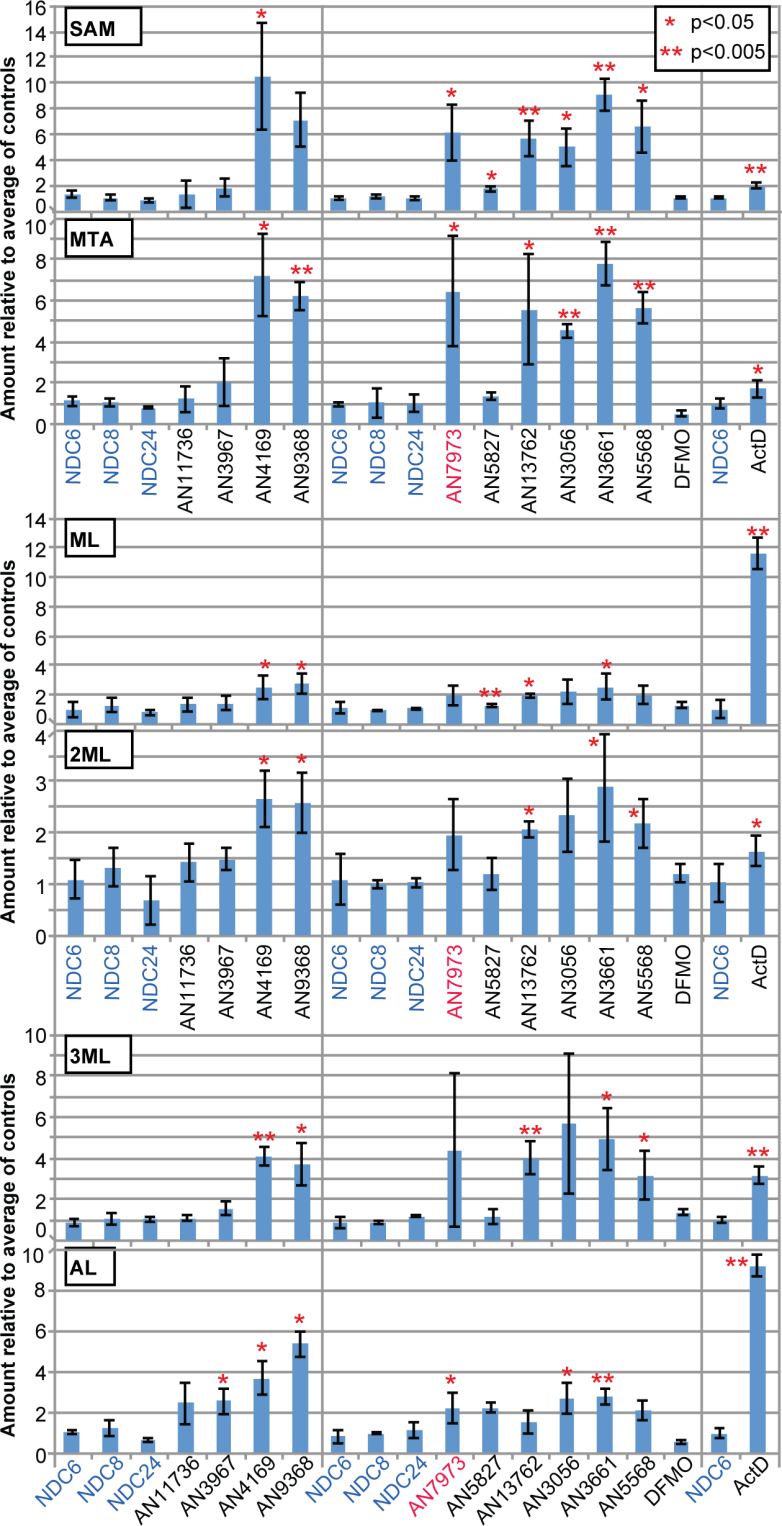
Effects of benzoxaboroles on the metabolome. Fold increases are shown for selected metabolites, relative to untreated cells (mean and standard deviation). The numerical data are in S6 Table. SAM: S-adenosylmethionine; MTA: methylthioadenosine; ML: methyllysine; 2ML: dimethyllysine; 3ML: trimethyllysine; AL: acetyllysine.

Another possible cause of splicing inhibition might be severe metabolic disruption. The fact that the parasites retained normal motility for several hours indicated that there were no immediate effects upon ATP generation, and we already knew that both mRNA transcription and *SLRNA* modification were unaffected. Analysis of the metabolome after 5h AN7973 treatment revealed, however, that the main effects were—as with AN5568—related to methylation, with substantial increases in S-adenosylmethionine (SAM) methyl thioadenosine (MTA), methyl lysine (ML), dimethyl lysine (2ML), trimethyl lysine (3ML) and acetyl lysine (AL) (Fig 5; S5 Table). SAM is a methyl donor and it has been shown that the methyl groups on the methylated lysines are derived from methionine [50]. It therefore seemed worthwhile to find out whether other anti-trypanosomal benzoxaboroles had similar effects.

**Fig 6:**
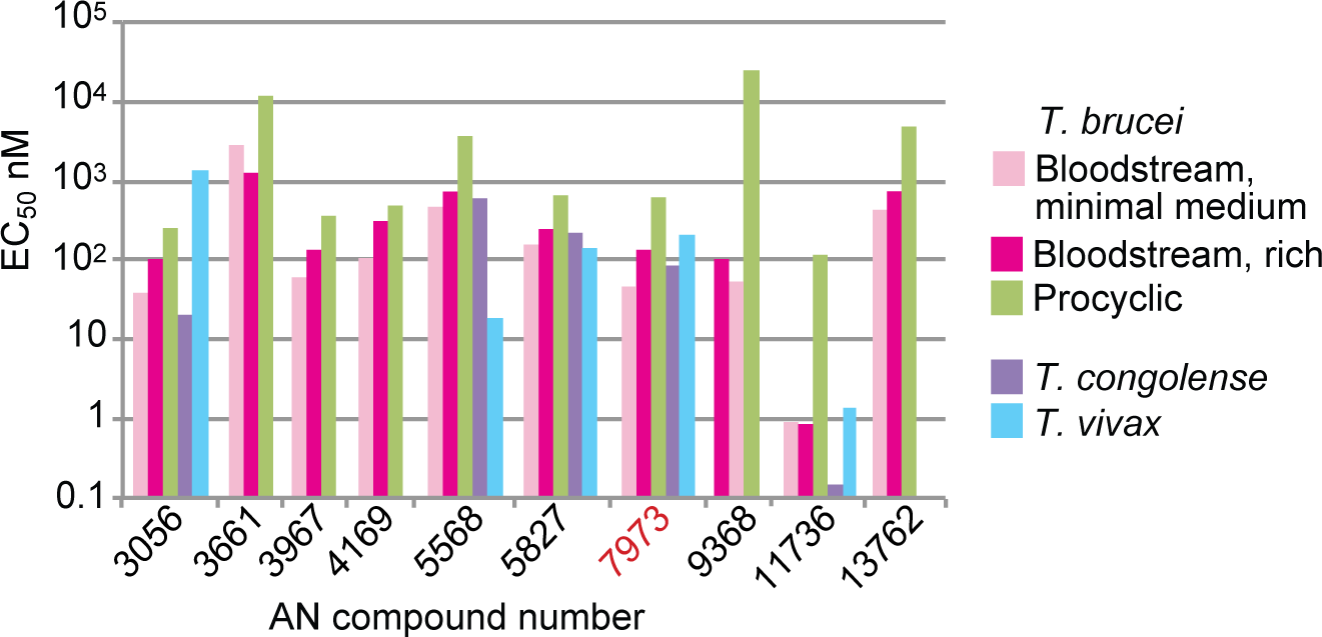
EC_50_s of selected benzoxaboroles for salivarian trypanosomes. The graph shows EC_50_s against *T*. *brucei* (in vitro, 48h assay for bloodstream forms, 72h for procyclic forms) and for selected compounds, also against *T*. *congolense* (*in vitro*) and *T*. *vivax* (*ex vivo*). Details for all tested compounds are in S1 Table.

We started with a panel of 30 compounds. These were tested *in vitro* against *T*. *brucei* and *T*. *congolense* (S2 Fig, S1 Table) and metabolic effects were also assessed (S6 Table). We then selected various compounds based on potency, the time taken to see an effect (S3 Fig), and the effects on methylated amino acids, SAM and MTA (S6 Table). Nearly all of the chosen compounds had EC_50_s in the low nM range (Fig 6). The notable exception was AN3661 (Fig 1), the anti-malarial lead compound that targets CPSF3 [26, 27]: its EC_50_ for bloodstream-form trypanosomes was at least 20 times higher than that of AN7973. At a concentration of 5x the EC_50_ determined at 48h, most of the compounds acted within 6-8h (S3 Fig, S1 Table). The veterinary drug candidate AN11736 kills the parasites more slowly because it requires processing for full activity (Giordani *et al*., manuscript in preparation). Results for treatment of *T*. *brucei* with the selected compounds are shown in Fig 5. There were no discernable structure-activity relationships for EC_50_, time to kill, or the SAM/MTA effect, but most of the oxaboroles that induced a SAM/MTA pattern were faster killing.

### Relationship between splicing inhibition and SAM/MTA accumulation

**Fig 7:**
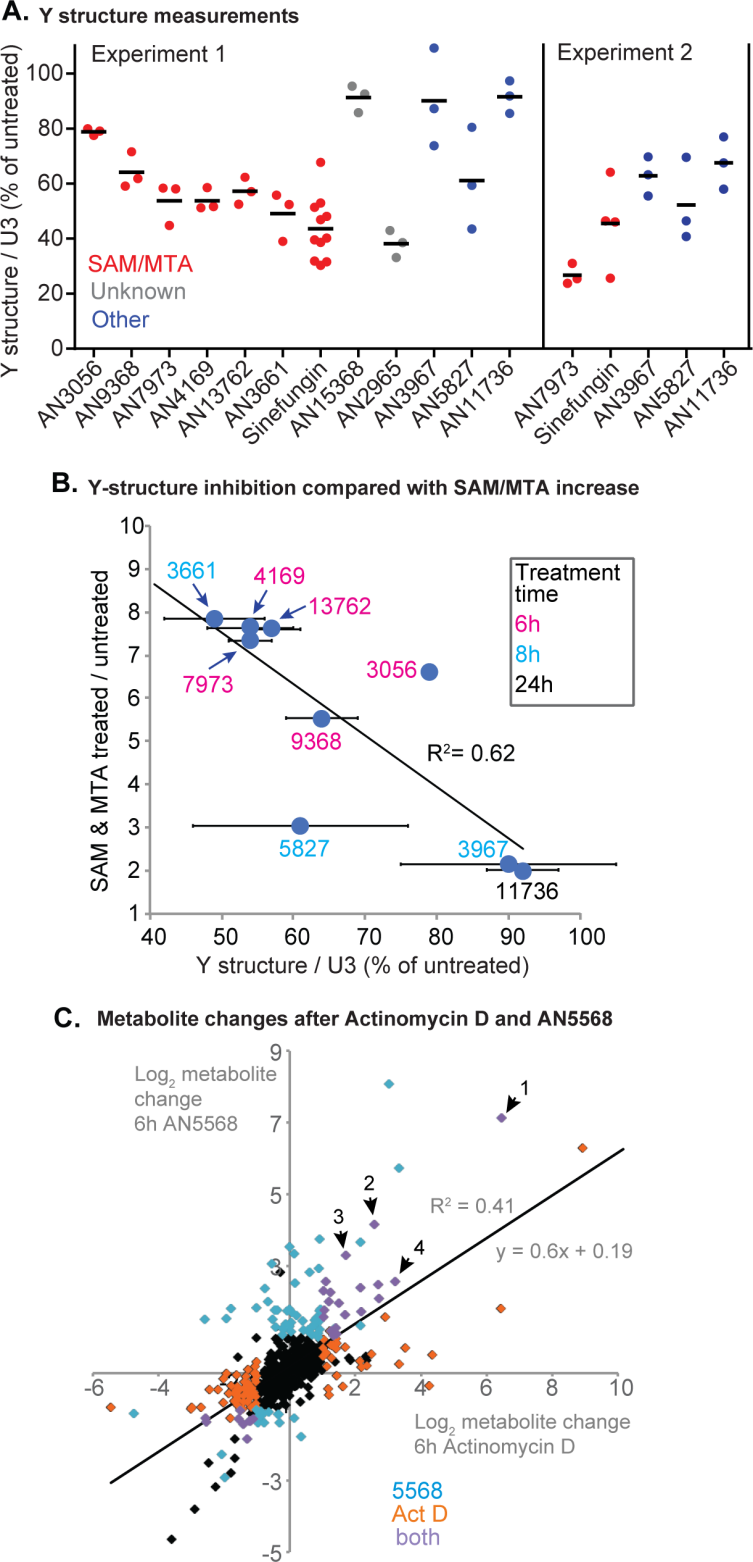
Relationship between *trans* splicing inhibition and methylation changes. A) Trypanosomes were incubated with 10x EC_50_ of various compounds for 2h, then splicing was assayed as in Fig 4. The % Y structure (relative to the U3 control) is shown. Results are shown for one experiment (experiment 1) with a full compound set and for a second experiment with selected compounds. A time course for the second experiment is shown in S4A Fig. B) The mean increase in SAM and MTA after treatment with each compound is plotted on the y-axis; the x-axis shows mean and standard deviation of the % Y-structure remaining (data for experiment 1 in (A)). The AN numbers are colour-coded to indicate the time of incubation prior to metabolome analysis. C. Metabolite changes after AN5568 or Act D treatment (both 5h). Each point represents a metabolite; data are in S7 Table. Orange: significantly affected by Actinomycin D; cyan: significantly affected by AN5568; purple: significantly affected by both treatments. Labelled metabolites are: 1- Glutathione disulfide; 2- Lys-Val-Pro; 3- N6,N6,N6-Trimethyl-L-lysine; 4- N6-Acetyl-L-lysine.

To investigate the possible link between splicing inhibition and the methylated metabolites, we measured the effects of the chosen compounds on splicing. We also included AN2965 (Fig 1), the compound whose mode of action had been investigated previously [22], and the antimalarial candidate AN13762 (Fig 1, compound 46 in [25]). Initial triplicate measurements (Fig 7A, experiment 1) gave reproducible results for most of the compounds; repeat assays for three that had given ambiguous results confirmed that they gave substantially less splicing inhibition than AN7973 (Fig 7, experiment 2 and S4A Fig). The clear processing inhibitors included the antimalarial candidate AN13762 (compound 46 in [25]); the EC_50_ of this compound for trypanosomes in the 3-day assay was 4 times higher than that for *P*. *falciparum*. Overall, there was a remarkably good correlation between splicing inhibition and the average increase in SAM and MTA (Fig 7B); only two compounds did not conform to the overall pattern. A caveat is that AN11736 is slow acting, and was used at low doses given its extreme potency, so it is possible that splicing inhibition by AN11736 might be seen upon more prolonged incubation.

Why could splicing inhibition and S-adenosyl methionine-related metabolites be linked? We could think of two explanations. First, transcription and processing of the *SLRNA* genes absorbs substantial cellular resources, since the cell needs to make at least 10,000 *SLRNA*s per hour [53]. It was possible that after 6-8h (the time of most metabolome measurements), there might have been some feedback inhibition of transcription that led to a decreased methylation requirement. Second, loss of mRNA production leads to loss of unstable mRNAs. If the protein encoded by an unstable mRNA has a high turnover rate, then that protein will disappear; and if that protein is a metabolic enzyme, its substrates will accumulate. This too could have led to metabolic changes as we noted.

To address the second hypothesis, we directly measured the effect of mRNA synthesis inhibition on the metabolome, using Actinomycin D (10 μg/mL) for 6h. Interestingly, there were again large increases in the amounts of methylated amino acids and significant (but smaller) increases in SAM and MTA as well (Fig 5). Comparison with the well-characterised effects of AN5568 revealed a clear correlation (Fig 7C, S7 Table). These results suggest that the increases in methylated and acetylated lysine—and perhaps also in SAM and MTA—that were seen after treatment with benzoxaboroles could indeed be an indirect consequence of loss of mRNA.

### *In vitro* splicing

As an alternative way to find out whether the effect of AN7973 on mRNA processing was direct or indirect, we measured *trans* splicing in permeabilised procyclic-form trypanosomes [48]. (Equivalent assays are not established for bloodstream forms.) As for most benzoxaboroles that have been tested (Fig 6, S1 Table) the EC_50_ of AN7973 for procyclic forms was 5–10 times higher than that for bloodstream forms. Treatment with 10x EC_50_ clearly inhibited splicing in procyclic forms, with 70% loss of Y structure after 2h (S4B and S4C Fig).

To test splicing *in vitro*, procyclic trypanosomes were permeablised with lysolecithin, pre-incubated with AN7973 or DMSO, then transcription was allowed to proceed for 10 min in the presence of [alpha-^32^P]-UTP [61]. RNA was separated on 7% polyacrylamide-urea gels and visualized by autoradiography. Under these conditions, the *SL* intron is visible as a ~100nt species, which disappears if incubation is continued for a further 20 min [61]; it is also not made if cap methylation is inhibited by S-adenosyl homocysteine [48].

**Fig 8:**
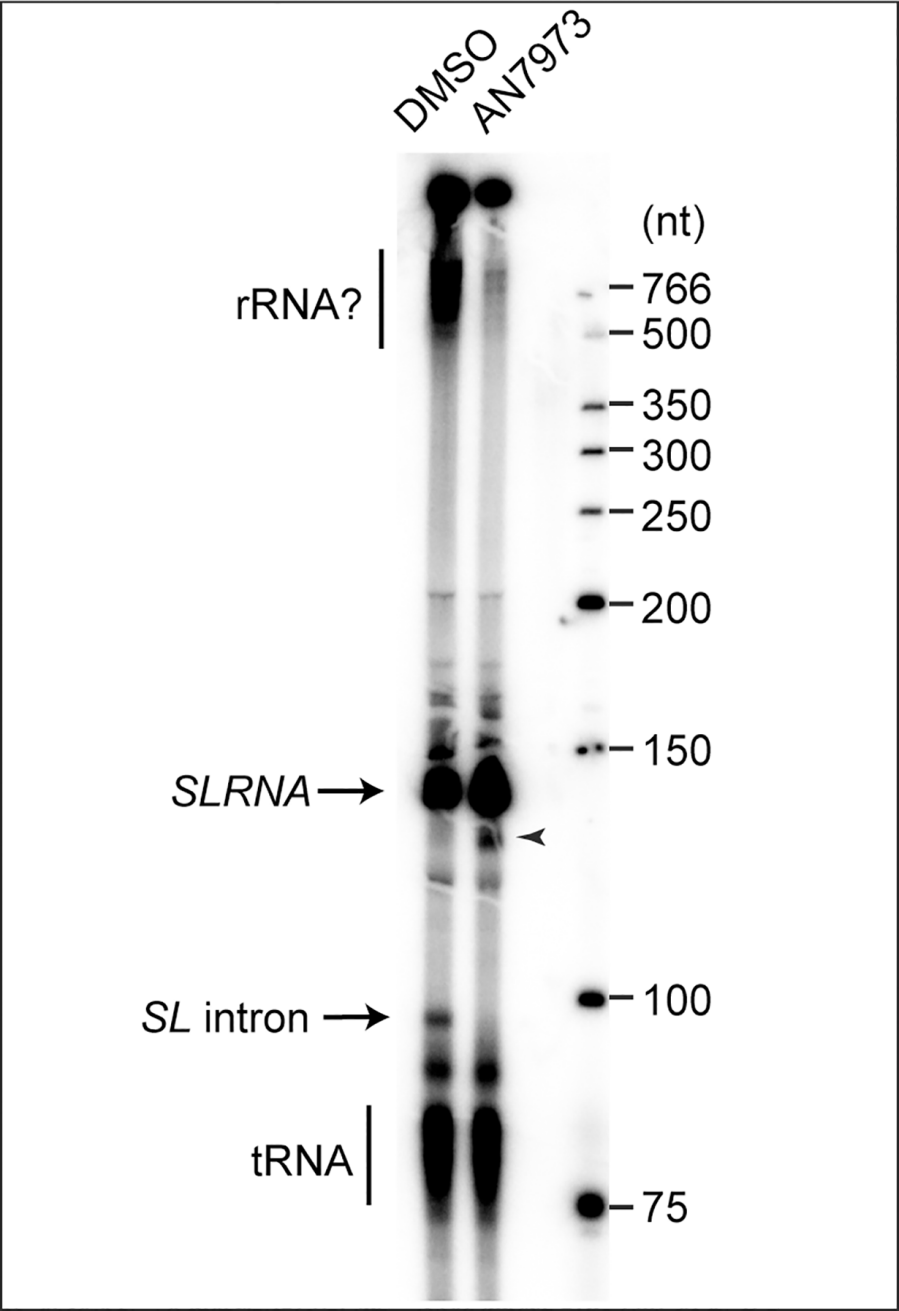
*In vitro* splicing assay. Permeablised procyclic-form trypanosomes (2.5 x 10^8^ / reaction) were incubated for 2 min in the presence of DMSO or AN7973, and then for 10 min in the presence of alpha-32P-UTP. Newly-made RNA was analysed by denaturing gel electrophoresis and autoradiography. The full-length SLRNA (~140nt) and the de-branched SLRNA intron (~100 nt) are indicated.

We do not know the intracellular concentration of AN7973, and in the *in vitro* transcription reaction the density of permeabilised parasites is 1000 times higher than in the experiments with cultures. For the *in vitro* assays we therefore chose to use a concentration of 100 μM (500x the EC_50_), which gives a AN7973:parasite ratio that is equivalent to the ratio at 5x EC_50_. This treatment reproducibly prevented formation of the Y structure without preventing transcription of *SLRNA* or smaller RNAs (Fig 8). An additional band (arrowhead), which appeared in the presence of AN7973 and was slightly shorter than *SLRNA*, might be a 3' degradation product that was previously described [48]. AN7973 also reproducibly inhibited labeling of RNAs longer than 500nt (indicated by a question mark). These RNAs are thought to include mRNAs and rRNA [62], and the inhibition could either be a consequence of *trans* splicing inhibition or another effect of AN7973. In future it would be interesting to repeat these studies at a variety of concentrations and with other benzoxaboroles.

### Benzoxaborole treatment induces formation of stress granules

**Fig 9:**
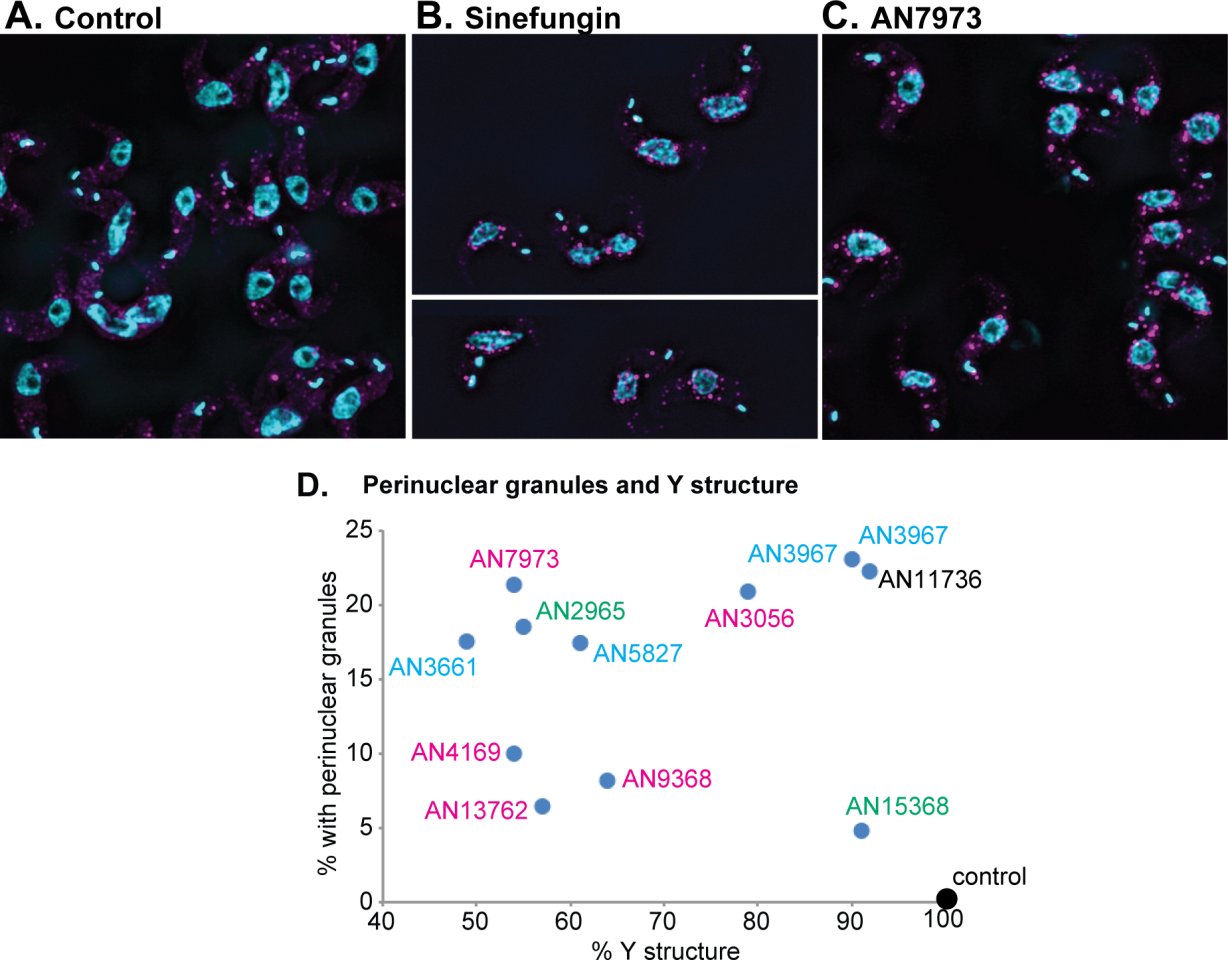
Effects of benzoxaborole treatment on formation of YFP-DHH1 granules. A-C) Bloodstream-form trypanosomes with one *DHH1* gene tagged *in situ* were washed with PBS, fixed with formaldehyde, then allowed to adhere to glass slides before imaging to detect YFP-tagged DHH1 (magenta) and DNA (cyan). Cells were (A) untreated, (B) incubated with Sinefungin (1μg/mL 30 min), or (C) treated with AN7973 (230 nM, 2h). D) Results for all tested oxaboroles. The average percentages of cells with clear peri-nuclear granules are plotted on the y-axis and the % Y-structure is on the x-axis. Times to show an effect on growth are as in Fig 6B. Results for the three replicate experiments are in S5 Fig and typical fields are shown in S6 and S7 Figs.

When trypanosomes are stressed by heat shock or starvation, their mRNAs accumulate in RNA-protein particles called stress granules containing a helicase, DHH1 [63]. Generally, these are throughout the cytoplasm, but after treatments that inhibit splicing, they transiently cluster around the nuclear envelope [64]. To find out whether AN7973 had the same effect we followed localization of YFP-tagged DHH1 in treated cells (Fig 9; the YFP signal is coloured magenta). A few granules were seen in untreated cells (Fig 9A) but after 30 min Sinefungin treatment, the nuclear periphery granule pattern was clear (Fig 9B). AN7973 also induced formation of perinuclear granules in a subset of cells (Fig 9C). However, when the remaining compounds were tested (S5 Fig, S6 Fig, S7 Fig) we observed no correlation between peri-nuclear granule formation and Y structure inhibition (Fig 9D). This may partly be explained by the transient nature of the perinuclear localization, but the clear perinuclear granule formation after AN11736 treatment also suggests that the pattern can be caused by stresses other than splicing inhibition. We concluded that without careful time-course studies, this assay could not be used to distinguish between specific splicing effects and more general stress responses.

### Ectopic expression of CPSF3 results in resistance to benzoxaborole splicing inhibitors

To try to identify possible targets implicated in mRNA processing, we re-examined the genomes of our partially-resistant lines (S2–S4 Tables). Since loss of polyadenylation stops splicing [36, 37], we looked for changes in genes associated with both processes. No mutations of the U snRNAs were found. A missense mutation was found in the Tb927.10.9660 open reading frame encoding a putative CRN/SYF3; this protein co-purified with the PRP19 complex, but did not co-sediment with the complex on a sucrose gradient [65]; its function is thus unclear. The Sm complex forms the core of spliceosomal snRNPs, and our two cell lines that had been selected in AN7973 both had 1–2 extra copies of the genes encoding four out of the seven components: Sm-B, Sm-E, Sm-F and Sm-G. The other three genes were, however, not amplified. After selection of proteins on an oxaborole affinity column, Jones et al. found enrichment of RBSR1 (Tb927.9.6870), a protein with an SR-domain that is potentially involved in splicing, and of the U2 splicing auxiliary factor (U2AF35, Tb927.10.3200) [22]. Our resistant lines had no changes in RBSR1 or U2AF35.

In *P*. *falciparum* and *T*. *gondii*, mutations in CPSF3 gave resistance to AN3661 [26, 27]. Moreover, in one of their AN2965 resistant lines, Jones *et al*. [22] observed a two-fold amplification of the gene encoding CPSF3 (Tb927.4.1340; also designated CPSF73); and AN2965 was a strong inhibitor of splicing (Fig 7A). We therefore compared the *T*. *brucei* sequence with those of *Plasmodium* and humans, concentrating on the residues that were mutated in AN3661-resistant *Apicomplexa*. H36 and Y408 of the *P*. *falciparum* sequence were mutated in AN3661-resistant lines but are retained as H and Y the human and trypanosome sequences (S8 Fig). The remaining important *Plasmodium* residues, however, are already different in trypanosomes. Y252 (mutated to C for AN3661 resistance) is N in *T*. *brucei*; T406 (mutated to I) is A; T409 (mutated to A) is C; and D470 (mutated to N) is already N in *T*. *brucei* (S8 Fig). From these changes alone one could predict that trypanosomes would be quite resistant to AN3661—as is indeed the case (S1 Fig). Although AN3661 did inhibit trypanosome mRNA processing and give a SAM/MTA effect, the concentrations used were 10–20 times higher than for AN7973 (S1 Table).

To test the role of CPSF3 we first attempted to make the equivalent of the Y408S mutation in trypanosomes by homologous gene replacement. Interestingly, although the transfections yielded several transgenic clones, none had the mutation. This, together with the fact that neither we nor Jones *et al*. [22] found the mutation after resistance selection, suggests that in the context of the trypanosome sequence, the Y408S equivalent (Y383S in the *T*. *brucei* sequence, S8 Fig) results in an unacceptable decrease in CPSF3 activity. If so, the result suggests either that the mutant CPSF3 has dominant-negative effects, or that CPSF3 is present in limiting amounts such that mutation of one gene copy results in haplo-insufficiency.

We next assessed how the differences between *T*. *brucei* and *P*. *falciparum* CPSF3 sequences would affect protein conformational dynamics. To do this we investigated a homology model of CPSF3 by elastic network normal mode analysis (S9A and S9B Fig), and found a relative rotational breathing motion of the domains (S9C Fig). Interestingly, in the model, many residues associated with AN3661 resistance, such as Y408 and N252 (*P*. *falciparum* numbering) were found to line the cleft between the breathing domains. Relative domain rotation might thus be able to enhance the accessibility of the binding site. Thus, mutations associated with AN3661 resistance might affect the conformational dynamics of the enzyme, the accessibility of the active site and the stability of the interdomain contacts.

**Fig 10:**
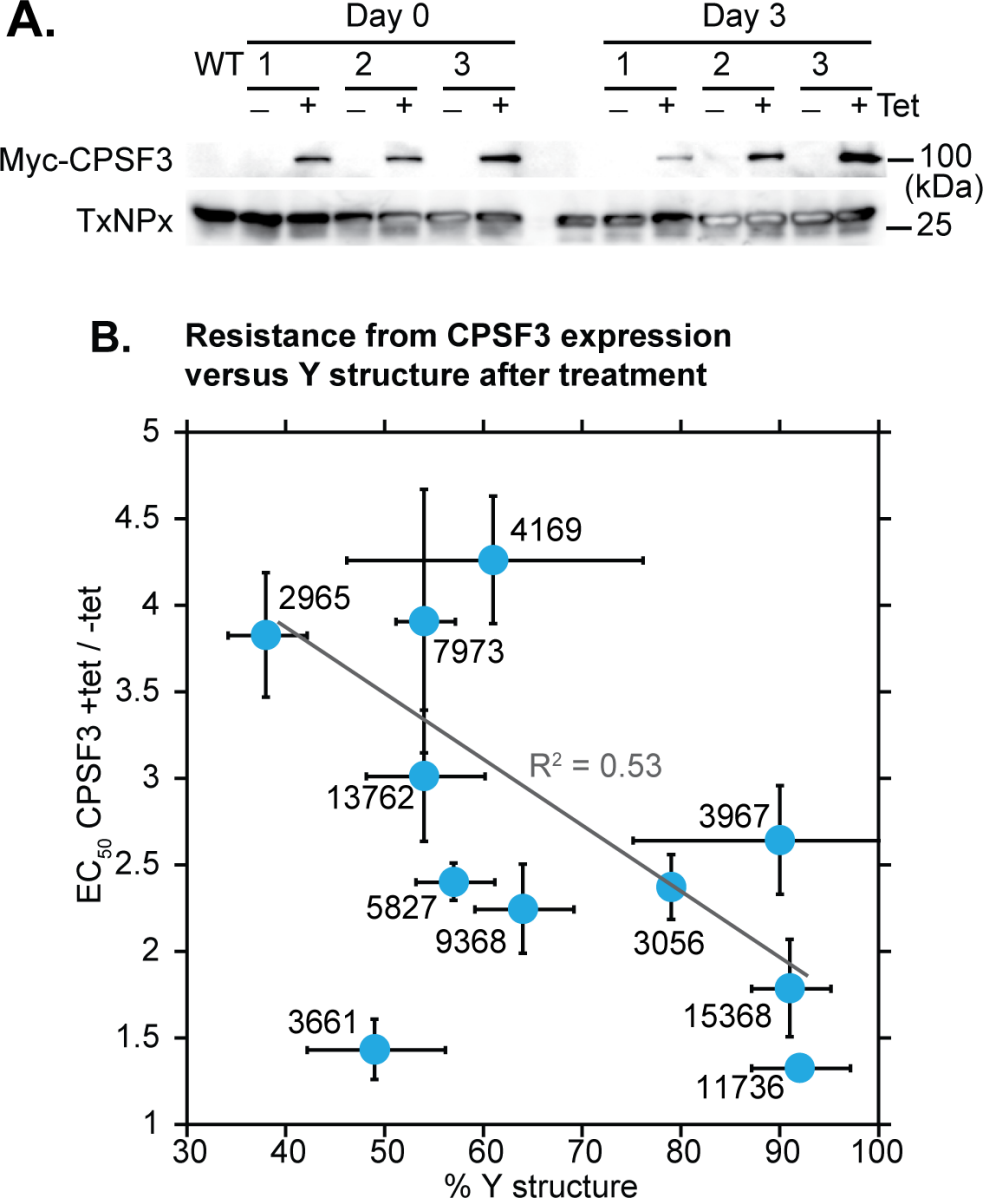
Ectopic expression of myc-tagged CPSF3 causes mild resistance to some benzoxaboroles. A) Expression of myc-CPSF3 in three independent cloned cell lines that were used for the assays. Expression was induced by tetracycline. Tryparedoxin peroxidase (TxNPx) served as a loading control. B) Correlation between the effect of myc-CPSF3 induction and the extent of Y-structure inhibition in the splicing assay. All results are arithmetic mean and standard deviation. Before plotting of the regression line and calculation of the correlation coefficient, the data for AN3661 were excluded because it was used at an extremely high concentration.

As an alternative to mutation, we inducibly over-expressed RBSR1, U2AF35 and CPSF3 in bloodstream forms, as C-terminally myc-tagged versions. Expression of RBSR1-myc and U2AF35-myc (S10A Fig) did not affect the EC_50_ of AN7973. In contrast, expression of CPSF3-myc caused at least 3-fold increases in the EC_50_s of four tested benzoxaboroles, including AN7973 (Fig 10, S10B Fig, S8 Table). Statistically significant increases of 2-fold or more were also seen for several other benzoxaboroles (Fig 10, S10B Fig, S8 Table) and there was a moderate correlation with Y-structure inhibition (Fig 10B). This suggests that the effective intracellular concentration of the benzoxaboroles is reduced through binding to excess CPSF3. The modest level of resistance may be explained by the fact that CPSF3 normally functions as part of a complex: we do not know the extent to which CPSF3 can be accumulated independently, and its conformation might be influenced by protein-protein interactions.

Over-expression of CPSF3-myc had almost no effect on the EC_50_ of AN3661. Because of its low potency, AN3661 was always tested at relatively high (micromolar) concentrations, so this result is difficult to interpret. However poor binding of AN3661 to CPSF3 is expected, since the trypanosome protein has residues equivalent to those found in the AN3661 resistant version of the *Plasmodium falciparum* protein (S8 Fig).

### Docking studies predict that inhibition of CPSF3 by AN7973 is feasible

To investigate whether AN7973 could interact with CPSF3, we performed induced fit docking to our comparative model of *T*. *brucei* CPSF3 (*Tb*CPSF3). For AN3661, it was proposed that boron occupies the position of an mRNA substrate phosphate group and that the ring oxygen, as well as hydroxylations, of the benzoxaborole can interact with the two active site zinc ions [27]. We applied constraints accordingly when docking AN7973 in the *Tb*CPSF3 active site. In our model, in addition to metal-oxygen interactions and hydrogen bonding contacts with D67, which stabilize the benzoxaborole binding mode, the pyrazole ring of AN7973 forms a hydrogen bond with the backbone amide nitrogen of A406 (S11 Fig). (All residue numbers mentioned correspond to those of the *P*. *falciparum* sequence, *Pf*CPSF3, S8 Fig) The elongated tail of AN7973 lodges in a subpocket that is largely lined by hydrophobic residues such as F267, P284, F286, L294, A406 and F443. Notably, the same interaction pattern was observed for both the tetrahedral form of the compound with a formal negative charge on the boron atom and the trigonal planar neutral variant (S11A and S11B Fig, respectively). The benzoxaborole core of the planar variant however has a rotated orientation compared to the tetrahedral form, which leads to a displacement of the loop containing Y408. The results of the docking therefore suggested that inhibition of *Tb*CPSF3 by AN7973 is feasible.

## Discussion

Benzoxaboroles are important drug candidates for both human and ruminant African trypanosomosis. Our results show that AN7973 inhibits mRNA processing in trypanosomes. Expression of additional CPSF3 increased the EC_50_ of AN7973, suggesting that AN7973 can bind to CPSF3. These results suggest that mRNA processing is an important target of AN7973, which might operate through CPSF3 inhibition. AN7973 also caused metabolite changes indicative of disturbed methylation, similar to those observed for acoziborole.

When this work was started, AN7973 was under consideration as a candidate for treatment of cattle trypanosomosis, but it was later found to be less effective against *T*. *vivax*. In the available *T*. *vivax* genome sequence there is a gap in the CPSF3 open reading frame. This gap spans N231 in the *T*. *brucei* sequence, which is Y in *Apicomplexa* and humans and mutated to C in resistant *Apicomplexa* (S8 Fig). All other CPSF3 residues that are implicated in AN3661 resistance in *Apicomplexa* are conserved between *T*. *vivax*, *T*. *congolense* and *T*. *brucei* (S8 Fig). Residues predicted to be in contact with AN7973 in our structural model of *Tb*CPSF3 were also largely conserved in the *T*. *vivax* sequence. Only in the loop 378–385 (or 403–410 in the *P*. *falciparum* numbering) are two valine residues replaced by isoleucine, which might slightly restrain the space accessible to the compound in the active site (S8 Fig).

Our results suggested a mechanistic link between splicing inhibition and accumulation of specific methylated metabolites (Fig 7B). One hypothesis we had was that the effects on methylation intermediates could be caused by decreased requirements for cap methylation. However we found no evidence, either *in vivo* or *in vitro*, that *SLRNA* transcription, capping or methylation were affected by AN7973. Another hypothesis was that loss of mRNA production will lead to a selective reduction in the activities of enzymes that have relatively high mRNA and protein turnover rates, and that these enzymes are required for various aspects of methylation. This was partially supported by accumulation of methylated amino acids after Actinomycin D treatment.

Strangely, our results revealed no clear structure-function relationships for benzoxaborole effects on trypanosome viability, metabolites, or splicing. The diversity in structures of compounds that inhibited trypanosome mRNA processing, combined with molecular modelling, suggests to us that this might be an intrinsic property of the pharmacophore. Nevertheless, some trends with respect to molecules with the same scaffolds can be noted. For example the two compounds AN15368 and AN11736 are both L-valinate amide benzoxaboroles [21] and both showed very little Y structure inhibition (Fig 1, Fig 7). These two compounds also showed little change in EC_50_ with overexpression of CPSF3 (Fig 10) suggesting that the L-valinate amide benzoxaborole series may not act through inhibition of mRNA processing. AN7973 and AN4169, both of the carboxamide scaffold, also showed similar decreases in Y structure. An important caveat is that the uptakes and metabolisms of the various compounds are likely to differ. For example, after 2h incubation, AN3056 and the veterinary drug candidate AN11736 had less effect on processing than did AN7973: but their action might be delayed, since both are subject to activation within the parasites.

So far, selection of trypanosomes resistant to benzoxaboroles has met very limited success. Substantial resistance was obtained only for compounds which require intracellular metabolic activation, and the mutations responsible were in the activating enzymes. For benzoxaboroles that are probably not metabolised, such as AN7973 and AN2965, only very limited resistance could be obtained. *A priori*, one would expect that if these compounds have a single active site target, selection for resistance should be relatively straightforward. It is however possible that the necessary mutations are incompatible with function.

Inhibition of mRNA processing was the earliest effect that was seen after AN7973 treatment and is therefore likely to make a vital contribution to parasite killing. In addition, both docking studies and results from over-expression identify CPSF3 as a likely target. This raises the possibility that similar modes of action might be seen in oxaboroles under development against other Kinetoplastids, including *Leishmania* and *Trypanosoma cruzi*.

## Materials and methods

### Ethical statement

All *in vivo* mouse experiments were carried out in accordance with the strict regulations set out by the Swiss Federal Veterinary Office, under the ethical approval of the Canton of Basel City, under license number #2813.

The experimental protocols used for goat studies were approved by the ethics committee for animal experimentation by the Veterinary Faculty of the University of Las Palmas in Gran Canaria, Spain on July 21, 2012 with the reference number 240/030/0121-36/2012. The studies were conducted under the strict guidelines set out by the FELASA for the correct implementation of animal care and experimentation.

Tests on cattle were done in accordance with the principles of veterinary good clinical practice (http://www.vichsec.org/guidelines/pharmaceuticals/pharma-efficacy/good-clinical-practice.html). The ethical and animal welfare approval number was 00 l-2013/CE-CIRDES.

### Compound efficacy studies for *T*. *congolense* and *T*. *vivax*

Testing of *T*. *congolense in vitro* (72 hrs) and *T*. *vivax ex vivo* (48 hrs) assays were done as described in [66]. Testing of *T*. *congolense* and *T*. *vivax in vivo* in mice was done as described in [21].

The proof of concept efficacy studies in goats using AN7973 were conducted as previously described in [67], but established and modified for *T*. *congolense* and *T*. *vivax* models of infection. The trials took place from January to May 2013 within the Veterinary Faculty of the University of Las Palmas and the Agricultural farm (Granja Agricola) of the Canarian Island Government in Arucas, Gran Canaria, Spain. In total, 45 female Canarian goats, weighing between 12–35 kg and no less than four months old, were purchased from a local dairy farmer and transported to the study site. The goats were placed in fly-proofed pens and allowed to acclimatise for two weeks, before being randomly selected and divided into test groups of four. Goats were experimentally infected intravenously from two highly parasitaemic donor goats, with 10^6^ and 10^5^ parasites per goat for *T*. *congolense* and *T*. *vivax*, respectively. AN7973 was administered intramuscularly accordingly, as either two injections of 10 mg/kg or as a single bolus dose of 10 mg/kg, on days 7 and 8 post-infection. Thereafter, the parasitaemia was monitored in the goats for up to 100 days post-treatment, after which any aparasitaemic and surviving goats were considered cured. Relapsed goats were removed immediately from the trial and humanely euthanized with an intravenous injection of sodium phenobarbital.

The efficacy of AN7973 against *T*. *congolense* and *T*. *vivax* in cattle was tested as described in [68]. The studies were conducted in fly-proof facilities and included negative (saline) controls; and the staff were blinded with regard to allocation of animals to treatment groups. Assessments were made for 100 days post treatment unless animals relapsed sooner.

### Trypanosome culture and compound treatment

Bloodstream-form *T*. *brucei brucei* 427 Lister strain were cultured in HMI-9 plus 10% foetal calf serum or CMM [69] plus 20% foetal calf serum at 37°C, 5% CO_2_. PCF *T*. *b*. *brucei* were cultured in SDM79 plus 10% foetal calf serum at 28°C. Compounds were dissolved at 20 mM in DMSO and aliquoted to avoid excessive freeze-thaw cycles.

EC_50_s were measured in two different ways. To obtain the EC_50_s in S1 Fig, compounds were serially diluted over 24 doubling dilutions in 100 μL culture medium in 96 well opaque plates from a starting concentration of 100 μM. Bloodstream form trypanosomes were added at a final density of 2x10^4^/mL (100 μL) and incubated for 48 hours, while procyclic forms were added at a final density of 2x10^5^/mL and incubated for 72 hours. After incubation, 20 μL of 0.49 mM Resazurin sodium salt in PBS was added to each well and plates were incubated for a further 24 hours. Plates were read on a BMG FLUOstar OPTIMA microplate reader (BMG Labtech GmbH, Germany) with λ_excitation_ = 544 nm and λ_emission_ = 590 nm.

For the results in S1 Table, sheet 1, column E, bloodstream form trypanosomes were diluted to 4000/mL in the presence of compounds (diluted in water from DMSO) and incubated for 72h. 3-4h before the end of the incubation, Resazurin (Sigma) was added (final concentration 44 μM). Resazurin fluorescence was measured to assess the number of surviving viable cells [70, 71]. Each assay was performed with 3 technical and 3 biological replicates.

For the time to kill assay (S1 Fig, S2 Fig) bloodstream-form *T*. *b*. *brucei* were cultured in 24 well plates in triplicate. Cultures were seeded at 5 x 10^5^/mL and compound was added at 5xEC_50_. Cells in each well were counted at 2, 4, 6, 8 and 24 hours using a haemocytometer.

For the all assays except the metabolomes shown in Fig 1 and S6 Table, compounds were used at 10x the 72-h EC_50_. To allow for variations between drug aliquots, EC_50_s were measured prior to every experimental series. The concentrations of compounds used in different experiments are listed in S1 Table and S6 Table.

### Cell cycle, protein and Northern blot analyses

For protein analysis, 2-3x10^6^ cells were collected for each sample, resuspended in Laemmli buffer heated and subjected to SDS-PAGE gel electrophoresis. All assays of macromolecular biosynthesis and RNA processing were done at densities of less than 2 x 10^6^/mL. Pulse-labeling was done as described in [72].

Total RNA was extracted from roughly 5x10^7^ cells using peqGold TriFast (peqLab) following the manufacturer's instructions. The RNA was separated on formaldehyde gels and then blotted on Nytran membranes (GE Healthcare). Following crosslinking and methylene blue staining (SERVA), the northern blots were hybridized with the appropriate probes. For mRNA detection, the membranes were incubated with [α-^32^P]dCTP radioactively labelled DNA probes (Prime-IT RmT Random Primer Labelling Kit, Stratagene) overnight at 65°C. For spliced leader detection, a 39mer oligonucleotide complementary to the spliced leader was labelled with [γ-^32^P]ATP using T4 polynucleotide kinase (NEB) and incubated with the membrane overnight at 42°C. After washing the blot, it was exposed to autoradiography films and detection was performed with FLA-7000 (GE Healthcare). The images were processed with ImageJ.

### Primer extension assays

Compound treatments were all done at cell densities of about 0.9x10^6^ cells/mL. For each condition, 8-10x10^7^ cells were used. Primer extension was done approximately as described in [47]; primers were: ACCCCACCTTCCAGATTC for *SLRNA* (KW01 or CZ6364) and TGGTTATTTCTCATTTAAGAGG (CZ6491) for U3 snRNA. Both primers and the ladder were radioactively 5'-end-labelled with [γ-^32^P]ATP. For extension, 10 μg of RNA was incubated for 5’ at 65° with 2 μL of dNTPs (10 mM) and roughly 200 000 counts per minute (cpm) of the corresponding primer. Afterwards, RNasin (Promega), SuperScript III Reverse Transcriptase (Thermo Fischer), DTT and buffer were added according to the manufacturers instructions. The mixture was incubated 60’ at 50°C and then inactivated 15’ at 70°C. The samples were run in 35 cm long 6% polyacrylamide gels, dried, and analysed by phosphorimaging. The images were analysed using Fuji / ImageJ.

### *In vitro* transcription and splicing

In vitro transcription in permeabilised cells was done following the published procedure [48, 61] with minor modifications [73]. Briefly, cells (2.5 x 10^8^/reaction) were permeabilised with lysolecithin for 1 min on ice, washed, then resuspended in 60 μL transcription buffer. 1 μL of either AN7973 or DMSO alone were added, and the reaction pre-incubated at 28°C for 2 min. After addition of 100 μL transcription cocktail containing 1 μL of either AN7973 or DMSO, the reaction was allowed to proceed for 10 min at 28°C. The permeabilised cells were pelleted (45 sec) and resuspended in 1 mL of TriFast. After the first phase separation, the aqueous fraction was re-extracted with phenol to remove residual protein; this was necessary to obtain good separation during gel electrophoresis. The final RNA volume was 20 μL. 10 μL of each reaction were separated on a 7% polyadcrylamide/urea gel and the products were detected by phosphorimaging with low molecular weight DNA markers (New England Biolabs).

### Genomic sequence analysis

For genomic DNA sequencing, libraries were prepared at the Cell Networks Deep Sequencing Core Facility (University of Heidelberg) and subjected to paired-end MiSeq (Illumina) at EMBL. The quality of sequencing was evaluated with FastQC and the reads were trimmed using Trimmomatic. The output was aligned to the *T*. *b*. *brucei* TREU927 genome (version 9.0) using bowtie2. Results are available in ArrayExpress under accession number E-MTAB-6307. The Picard option AddOrReplaceReadGroups was used to create a valid .bam file to then be piped into GATK for obtaining, as output, .vcf files containing SNP and indel information. SnpSift filtered the features of interest, excluding for example synonymous mutations and intergenic regions. Identified variations from all cell lines were pooled to look for mutations found in all strains compared to the wild type, and in addition, reads from each strain were processed separately to find all the mutated genes. The lists were then compared. The genes taken into consideration were identified with annotation and categories from the Clayton lab in-house annotation list. Based on these annotations, the genes were filtered in Excel, where highly repetitive genes were excluded from the analysis. At first, variant surface glycoproteins (VSG), expression site-associated genes (ESAG), receptor-type adenylate cyclase GRESAG, UDP-Gal or UDP-GlcNAc-dependent glycosyltransferases and pseudogenes were removed. In the case of non-homozygous mutations, the genes were further selected filtering out other repetitive genes such as leucine-rich repeat proteins (LRRP), various invariant surface glycoproteins (ISG), nucleoside transporters (TbNT) and retrotransposon hot spot (RHS) proteins. The list of gene IDs was compared with their translation level based on ribosome profiles [74, 75] and genes with values below 10 (non-translated) were excluded from the analysis.

### Metabolomics

For all assays at 5x EC_50_, bloodstream-form *T*. *b*. *brucei* were inoculated into medium at 1 x 10^6^/mL (for a six or eight hour incubation) or 2 x 10^5^/mL (for a 24 hour incubation) and compounds were added. Cells were incubated with compounds under normal growth conditions. At the desired time point, 1 x 10^8^ cells were taken and cooled rapidly in a dry-ice ethanol bath to 4°C. Samples were centrifuged at 1250 g twice to remove all medium before 200 μL chloroform:methanol:water (1:2:1) were added. Extracts were shaken for one hour at max speed before cell debris was removed by centrifugation at 16,000 g. Metabolite extracts were stored at -80°C under argon gas.

*Mass spectrometry–*Metabolite samples were defrosted and run on a pHILIC column coupled to an Orbitrap mass spectrometer as previously described [76]. In batch 1 an Orbitrap Exactive (Thermo Scientific) was used with settings including mass range: 70–1400, a lock mass of 74.0964, capillary: 40V, Tube lens: 70V, Skimmer: 20V, Gate lens: 6.75V and C-trap RF: 700V. In batch 2 an Orbitrap QExactive (Thermo Scientific) was used with settings including mass range: 70–1050, lock masses of 74.0964 and 88.0757, S-lens: 25V, Skimmer: 15V, Gate lens: 5.88V and C-trap RF: 700V. For fragmentation analysis an MS2 isolation window of 4 m/z, an intensity threshold of 3.3e5 and dynamic exclusion of 10 seconds were used.

Metabolites were putatively annotated using IDEOM software [77] before verification of annotations using mass, retention time, isotope distribution and fragmentation pattern. Xcalibur (Thermo) was used to explore the raw data, MzCloud (mzcloud.org) was used to match fragments to database spectra. Metabolite analysis was done using four biological replicates per condition and cell line. Relative metabolite levels were based on raw peak height relative to the average raw peak height of untreated cells. Lists of metabolites were mapped on metabolic pathways using Pathos (http://motif.gla.ac.uk/Pathos/). Intersections between samples were found using bioVenn (http://www.cmbi.ru.nl/cdd/biovenn/). (http://www.cmbi.ru.nl/cdd/biovenn/). Metabolite identities are consistent with standards from the Metabolomics Standards Initiative and evidence for each identity is shown in S6 Table.

### YFP-DHH1 and expression of myc-tagged proteins

For over-expression of C-terminally myc-tagged proteins, the open reading frames encoding proteins of interest were amplified by PCR from genomic DNA, and cloned into pRPa-6xmyc [78]. After transfection, cells were selected and expression of myc-tagged protein was induced overnight with 100 ng/mL tetracycline.

The plasmid for creation of cells expressing YFP-DHH1 [59] was a kind gift from Susanne Krämer (University of Würzburg). It was transfected into bloodstream-form trypanosomes and two stable cell lines expressing the protein were selected. Trypanosomes (maximum density less than 1 million/mL in 10 mL) were treated for 30 min with 2 μg/mL Sinefungin, or for 1-2h with compounds at 10x EC_50_. After collection (5 min 1000g) and washing in PBS cells were resuspended in 20 μL PBS. 500 μL of 4% paraformaldehyde solution in PBS was added, cells were incubated without shaking for 18 min, washed 3x with PBS, then distributed onto poly-lysine coated chamber glass slides (all cells divided in two chambers) and left at 4°C over night. The PBS was then removed and cells were permeabilised using 0.2% Triton X-100 (w/v) in PBS, with shaking at room temperature for 20 min. After a further 3 washes, slides were incubate at room temperature with 200 ng/mL DAPI in PBS (15 min shaking), washed twice more, air-dried, embedded and covered for microscopy.

Slides were viewed and the images captured with Olympus IX81 microscope. The 100x oil objective was used. Digital imaging was done with ORCA-R2 digital CCD camera C10600 (Hamamatsu) and using the xcellence rt software. Bright field images were taken using differential interference contrast (DIC), Exposure time 30 ms, Lamp 4.0. Fluorescent images were made using DAPI and YFP filters. They were taken as Z-stacks, 30–40 images in a 8 μm thick layer, Exposure time 40 ms, Light intensity 100%, and afterwards they were deconvoluted (Numerical aperture 1.45, Wiener filter, Sub-Volume Overlap 20, Spherical Aberration Detection Accurate).

The fluorescence images were processed using ImageJ. For YFP and DAPI channels, layers containing signals ere selected, then the projection of maximum intensity of the deconvoluted stack was used. The images were saved in an 8-bit range then overlayed with DAPI in cyan and YFP as magenta. The colour balance was then adjusted with variable maxima for DAPI/cyan, but a set maximum of 80 for YFP/magenta.

After initial assessment after AN7973 and Sinefungin treatment, three independent experimental series, each including a negative control, were processed, and the images were read blinded. Cells were classified as having nuclear periphery granules if there were at least four strong granules on top of, or within one granule diameter of, the nucleus. Cells were classified as "strong" if nearly all granules were around the nucleus, and "possible" if there were several granules in other positions as well. For granule counts, only structures with at least four adjacent pixels at maximum intensity were considered.

#### Homology modeling

Models of *Trypanosoma brucei* (*Tb*CPSF3) and, for comparison, *Plasmodium falciparum* CPSF3 (*Pf*CPSF3), were built with the Advanced Homology Modeling routine of Schrödinger Maestro [79]. A BLAST search [80] was performed and the crystal structure of human CPSF73 with PDB ID 2i7v was chosen as the template for both models (Resolution 2.1 Å; sequence identity 56% and 43% for TbCPSF3 and PfCPSF3, respectively). Globally conserved residues in the beta-CASP family were identified from a multiple sequence alignment making use of the Pfam database [81]. Conformations of the globally conserved residues E293, L298 and G386 (*P*. *falciparum* numbering) were retained similar to the template structure without further modification. Alignment of template and query sequences was performed by ClustalW. A knowledge-based model, including the template structure-Zn^2+^ ions, was built and subjected to protein preparation without further loop refinement. Addition of hydrogen atoms and bond order assignment were done with the Protein Preparation Wizard of Maestro [79]. Amino acid protonation states at pH 7.0 were determined using PROPKA [82]. Next, the hydrogen bonding network was optimized and models were subjected to a restrained energy minimization using the OPLS3 force field allowing for a maximum heavy atom RMSD of 0.3 Å [83]. For validation of the structural models, PROCHECK and QMEAN analyses were performed [84]. Results are summarized in S9 Table. The analysis demonstrates that the models are of reasonable quality and that, in particular, the binding site close to the zinc ions, as the main region of interest, showed good local quality (S12 Fig).

#### Normal mode analysis

The NOMAD-Ref webserver was used to calculate normal modes for the modeled structure of TbCPSF3 using an elastic network model (ENM) and considering all atoms [85]. 16 modes were calculated with a distance weight parameter of 5.0 Å for the elastic constant, an ENM cutoff of 10 Å and an average RMSD of 1.0 Å in output trajectories, using the automatic method.

#### Compound preparation and docking studies

AN7973 was considered in the neutral, trigonal planar form, and as the conjugate Lewis base with a formal negative charge on the boron and tetrahedral geometry [8, 86, 87]. Since a non-substituted benzoxaborole has a pKa of 7.3, a non-substituted 3,3-dimethyl benzoxaborole a pKa of 8.3 and, depending on nature and position of a substitution on the benzene ring, further shifts towards a more basic or acidic pKa were possible, the preferred geometry of AN7973 was unclear [86]. Moreover, a possible influence of the positively charged metal ions in the protein binding site on the compound state could not be excluded.

Docking studies were performed for the models of TbCPSF3 and PfCPSF3. We used the Induced Fit Docking protocol of Schrödinger, since the template structure for the homology modeling did not have any bound ligands and it is thus likely that ligand binding requires some reorganization in the binding site [88–90]. The standard protocol to produce up to 20 receptor-ligand complexes per ligand and the OPLS3 force field were chosen [83]. Grids were centered on Y408 or the respective equivalent (PfCPSF3 numbering) and had a size of 26 Å in x-, y- and z-directions. Docking results were constrained by requiring a ligand contact with each of the two zinc ions: any acceptor atom of the ligand within a distance no greater than the sum of the van-der-Waals radii of zinc and the respective ligand atom plus an additional 0.4 Å satisfied the constraint. Ligand ring conformations were sampled and discarded if their energy was more than 2.5 kcal/mol higher than the conformation lowest in energy. Non-planar conformations of amide bonds were penalized and the torsional potential around bonds which should adopt a planar geometry was increased to lessen the likelihood of observing non-planar geometries. In the initial Glide docking step, the van-der-Waals radii of both the receptor and ligand atoms were scaled by a factor of 0.50 to mimic some flexibility. A maximum number of 20 poses per ligand was collected at this stage and subjected to a Prime refinement. Residues within 5 Å of the initial ligand pose were refined and side chain optimization was performed. Finally, redocking was carried out using the standard precision protocol to dock the ligands into all refined protein structures within 30 kcal/mol of the best structure and the top 20 structures overall.

## Supporting information

S1 Table EC_50_s and compound concentrations used in different experimental series. For a detailed legend see the top sheet.
(XLSX)

S2 Table Summary of copy number changes, single nucleotide polymorphisms (snps) and insertions and deletions (indels) in cells with weak resistance to AN7973. A description of the experiments is on sheet 1.
(XLSX)

S3 Table Details of snps and indels in cells with weak resistance to AN7973. For a detailed legend see the top sheet.
(XLSX)

S4 Table Details of copy number variations in cells with weak resistance to AN7973. For a detailed legend see the top sheet.
(XLSX)

S5 Table Metabolite changes after treatment of wild-type and mildly resistant cells with AN7973 for 5h. For a detailed legend see the top sheet.
(XLSX)

S6 Table Levels of chosen metabolites after treatment with different compounds. For a detailed legend see the top sheet.
(XLSX)

S7 Table Metabolomic effects from treating trypanosomes with Actinomycin D or AN5568 for 5h. (XLSX)

S8 Table EC50s of various benzoxaboroles in cells with and without additional CPSF3 expression. (XLSX)

S9 Table Global validation of homology models. Details are in the Table.
(XLSX)

S1 Fig Statistical analysis of results in Fig 3. A: Results for an initial experiment in which the level of Y structure was quantified relative to *SLRNA*. S 30' is the control value after incubation with Sinefungin for 30 min.
B—F: 95% confidence intervals of mean differences between Y structure quantifications for panel A and for Fig 3E–3H. The intervals were calculated using Dunnet’s multiple comparisons test. In each case the calculation is done for the comparison between no treatment and the treatment indicated. The higher the difference, the lower the chance that the values were not significantly different. A value of 0 indicates a 5% chance that differences were not significant. Sinefungin values are for the difference between no drug and 30 min with Sinefungin.
(PDF)

S2 Fig A. Growth curves of bloodstream-form *T. brucei* in the presence and absence of benzoxaboroles. (PDF)

S3 Fig Time-to-kill assays for benzoxaboroles—like S1 Fig but with more compounds and without the untreated control. (PDF)

S4 Fig Effects of benzoxaboroles on Y structure levels. A. Time course of effects of AN7973, AN3967, AN5827 and AN11736 in bloodstream forms (Experiment 2 in Fig 7). Sinefungin treatment was for 30 min.
B, C: effect of AN7973 in procyclic forms.
(PDF)

S5 Fig Results for three separate experiments (A, B and C) in which the location of YFP-DHH1 was analysed. The average percentages of cells with clear (dark blue) or possible (pale blue) peri-nuclear granules are plotted as bars, with the average numbers of large granules (at least 4 contiguous pixels at maximum intensity) anywhere in the cell displayed as black spots. The dotted line corresponds to the negative control.
(PDF)

S6 Fig YFP-DHH1 localization after treatment with different benzoxaboroles. These are not "typical" images; instead, fields in which nuclear periphery granules were present have been chosen. Some (but not all) examples of peri-nuclear granule patterns are indicated by arrows. Compounds used are shown on each image. For AN7973 examples from the three different experiments are shown. The key is on the bottom left.
(TIF)

S7 Fig YFP-DHH1 localization after treatment with different benzoxaboroles. These are not "typical" images; instead, fields in which nuclear periphery granules were present have been chosen. Compounds used are indicated and the key is as in S6 Fig. The control image in this case had brighter YFP fluorescence, for unknown reasons.
(TIF)

S8 Fig Sequences of CPSF3 from *T. brucei* (TREU927), *T. congolense, T. vivax, Plasmodium falciparum* and *Homo sapiens*. Only the conserved region is shown. Black-highlighted residues are identical in at least four sequences and blue ones indicate conservative replacements. For key to symbols and numbers see the bottom of the Fig. The gap in the *T*. *vivax* sequence is present because of a gap in the genome assembly.
(PDF)

S9 Fig Domain annotations, residues linked to resistance mutations in *P. falciparum* and *T. gondii* mapped onto a structural model of *Tb*CPSF3 and exemplary results of the normal mode analysis for the *Tb*CPSF3 structure. A. Conserved domains found in CPSF3 adapted from the Pfam domain annotations. RMMBL: Zn-dependent metallo-hydrolase RNA specificity domain; CPSF73-100_C: C-terminal conserved region of the pre-mRNA 3'-end-processing of the polyadenylation factor CPSF-73/CPSF-100 proteins. As indicated, this C-terminal domain is not present in the structural model.
B. Homology model of *Tb*CPSF3 in cartoon representation colored according to the domain annotations in (A). Residues associated with resistance to benzoxaboroles are highlighted as sticks; the residue numbering corresponds to the *P*. *falciparum* sequence. Grey dots indicate the predicted binding pocket for AN7973 for reference.
C. Overlay of two snapshots from the first (slowest) mode obtained by normal mode analysis of the *Tb*CPSF3 model structure. The protein is shown in ribbon representation and colored according to the domain annotations in (A). The modes show a general relative rotational breathing motion of the domains as indicated by the arrows. Two important residues lining the interdomain contact region, N252 and Y408 (*P*. *falciparum* numbering) are shown as red sticks for reference.
(PNG)

S10 Fig Details of EC_50_ results in cells with and without CPSF3-myc expression. (A) Induced expression of Myc-tagged RBSR4 and U2AF35.
(B) IC_50_ measurements in cells with tetracycline-inducible expression of myc-CPFS3.
(PDF)

S11 Fig Induced fit docking results for AN7973 in the active site of TbCPSF3. AN7973 (cyan sticks) was docked in the negatively charged form (A, tetrahedral geometry) and the neutral form (B, trigonal planar geometry). In both cases, the top scoring poses with Glide docking scores of -9.3 kcal/mol and -8.4 kcal/mol, respectively, are shown. For the tetrahedral geometry, in total, two poses with an average docking score of -9.0±0.4 kcal/mol and for the planar geometry, three poses with an average docking score of -6.9±1.4 kcal/mol were obtained. The TbCPSF3 homology model is shown in grey cartoon representation with important interacting residues highlighted as sticks and the zinc ions as grey spheres. Dashed lines indicate metal-coordination bonds (grey), metal-ligand interactions (black) and hydrogen bonds to the ligand (purple). The residue numbers correspond to the *Plasmodium falciparum* CPSF3 sequence.
(PNG)

S12 Fig Local quality estimate by QMEAN score mapped to the protein structures of hCPSF73 (template) and the homology models of *Tb*CPSF3 and *Pf*CPSF3. Proteins are shown in cartoon representation. The location of the binding site is indicated by the zinc ions, shown as grey spheres. Local quality estimates are color coded from red (worst) to blue (best).
A. human template structure 2i7v, hCPSF73; B. *Tb*CPSF3; C. *Pf*CPSF3.
(PNG)
