# Supplementary material for: The trypanocidal benzoxaborole AN7973 inhibits trypanosome mRNA processing
Source: PLoS Pathog. 2018 Sep 25;14(9):e1007315. doi: 10.1371/journal.ppat.1007315 (PMC6173450; doi:10.1371/journal.ppat.1007315)
Supplement: S1 Fig — A: Results for an initial experiment in which the level of Y structure was quantified relative to SLRNA. S 30' is the control value after incubation with Sinefungin for 30 min. B—F: 95% confidence intervals of mean differences between Y structure quantifications for panel A and for Fig 3E–3H. The intervals were calculated using Dunnet’s multiple comparisons test. In each case the calculation is done for the comparison between no treatment and the treatment indicated. The higher the difference, the lower the chance that the values were not significantly different. A value of 0 indicates a 5% chance that differences were not significant. Sinefungin values are for the difference between no drug and 30 min with Sinefungin. (PDF) [file ppat.1007315.s010.pdf]

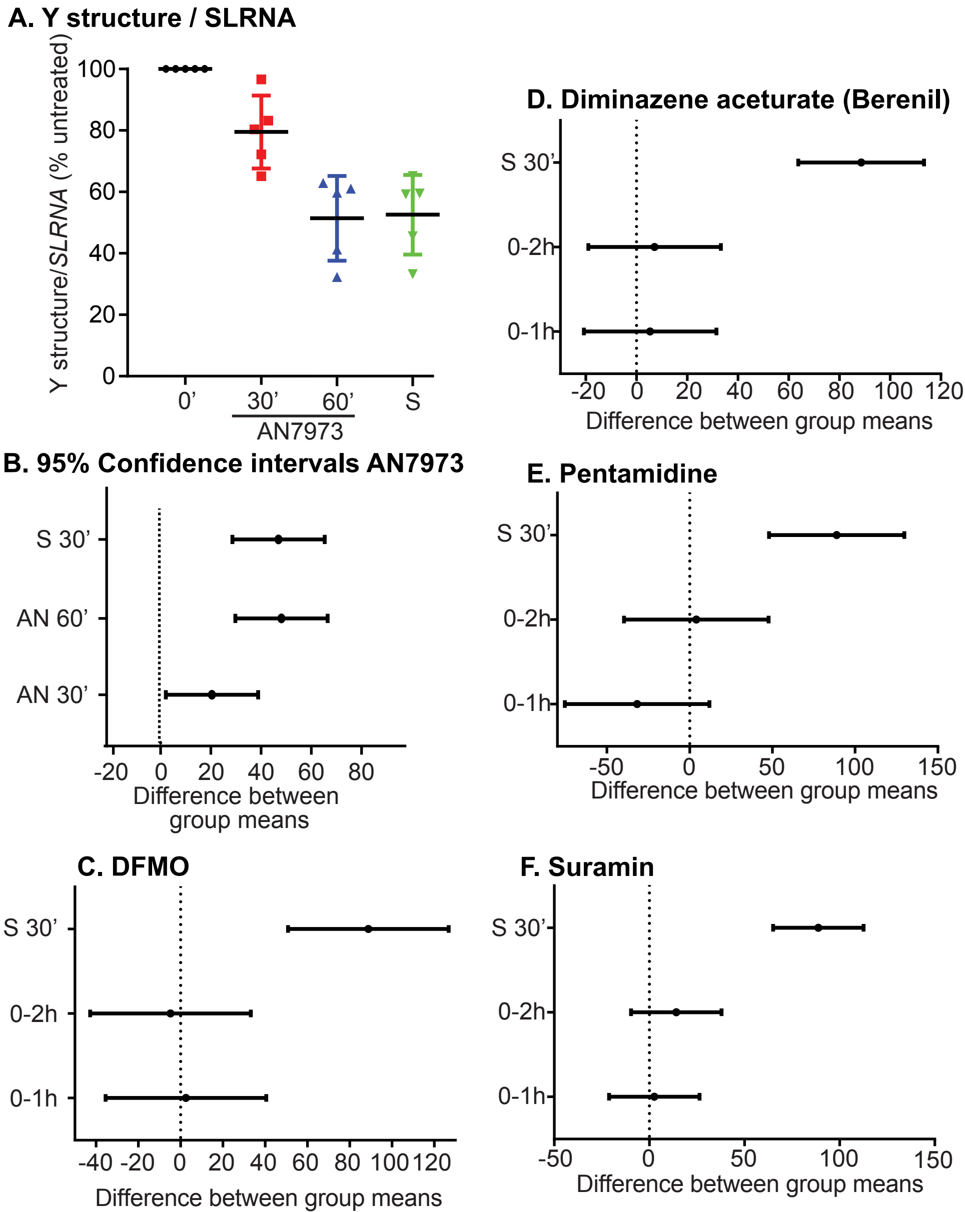

A. Y structure / SLRNA
Y structure/SLRNA (% untreated)
100
80
60
40
20
0
0'
30'
60'
S
AN7973
B. 95% Confidence intervals AN7973
S 30'
AN 60'
AN 30'
-20
0
20
40
60
80
Difference between group means
C. DFMO
S 30'
0-2h
0-1h
-40
-20
0
20
40
60
80
100
120
Difference between group means
D. Diminazene aceturate (Berenil)
S 30'
0-2h
0-1h
-20
0
20
40
60
80
100
120
Difference between group means
E. Pentamidine
S 30'
0-2h
0-1h
-50
0
50
100
150
Difference between group means
F. Suramin
S 30'
0-2h
0-1h
-50
0
50
100
150
Difference between group means
