# Supplementary figures and images for: The trypanocidal benzoxaborole AN7973 inhibits trypanosome mRNA processing
Source: PLoS Pathog. 2018 Sep 25;14(9):e1007315. doi: 10.1371/journal.ppat.1007315 (PMC6173450; doi:10.1371/journal.ppat.1007315)

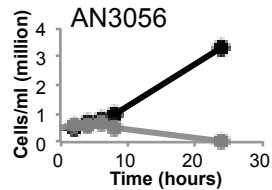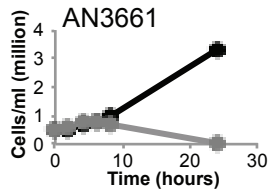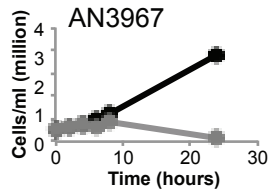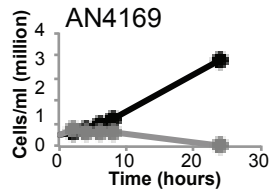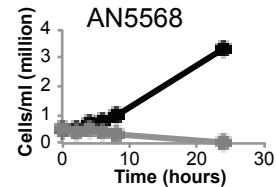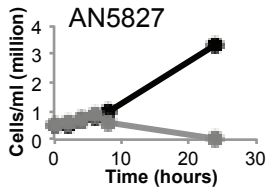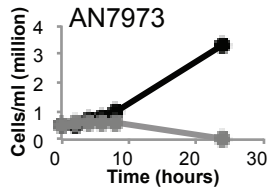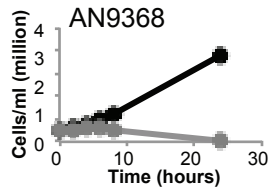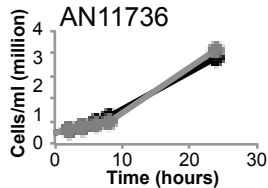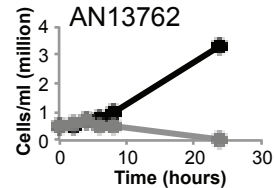

Supplement: S2 Fig — (PDF) [file ppat.1007315.s011.pdf]

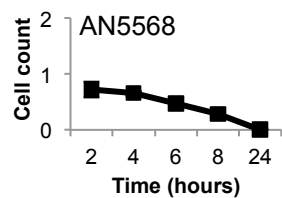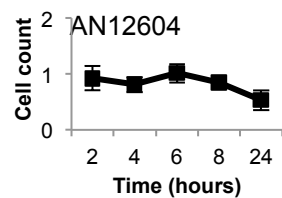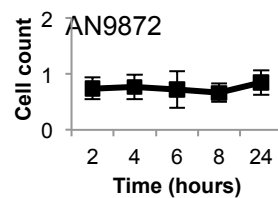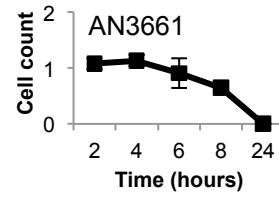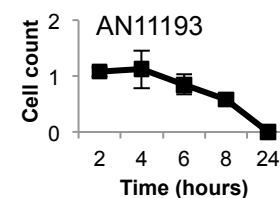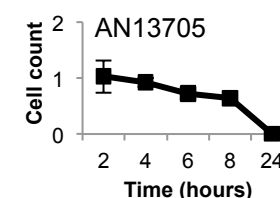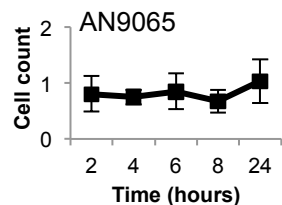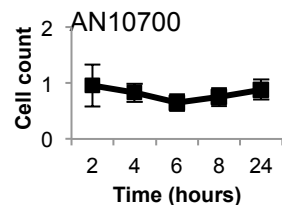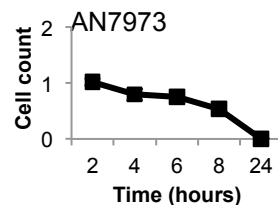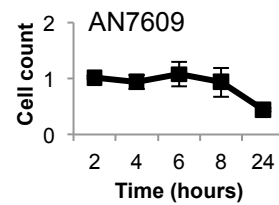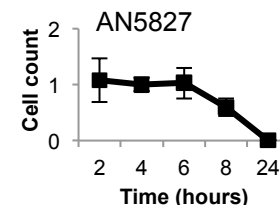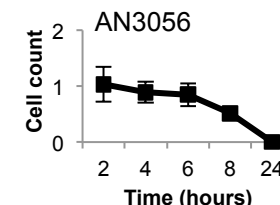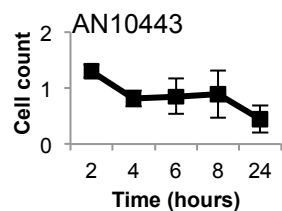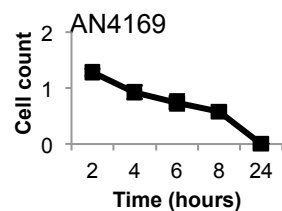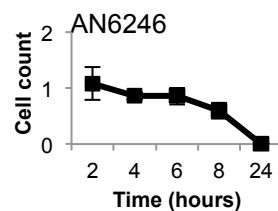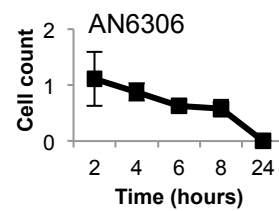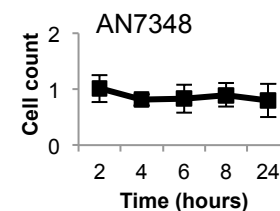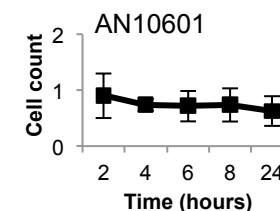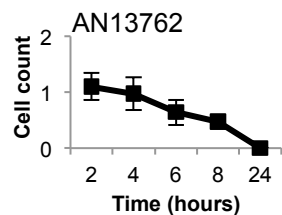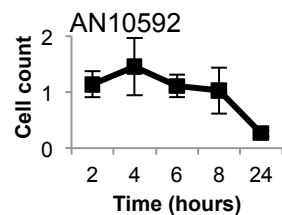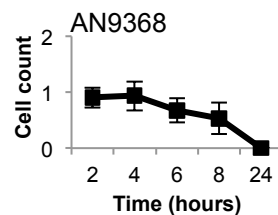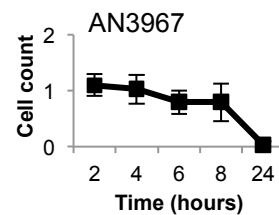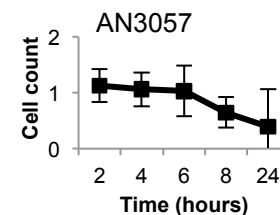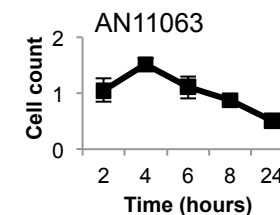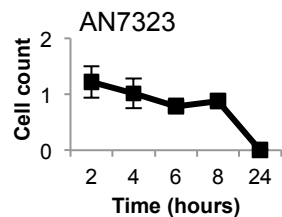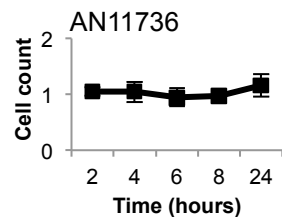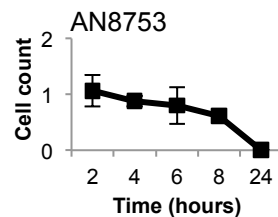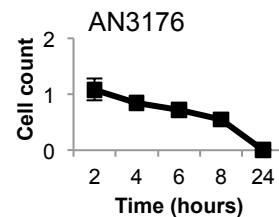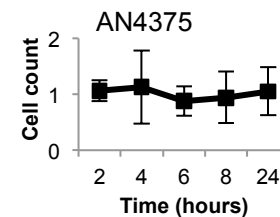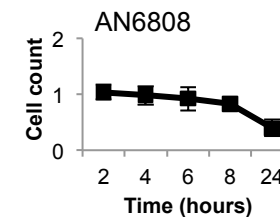

Supplement: S3 Fig — (PDF) [file ppat.1007315.s012.pdf]

## A. Time course, experiment 2

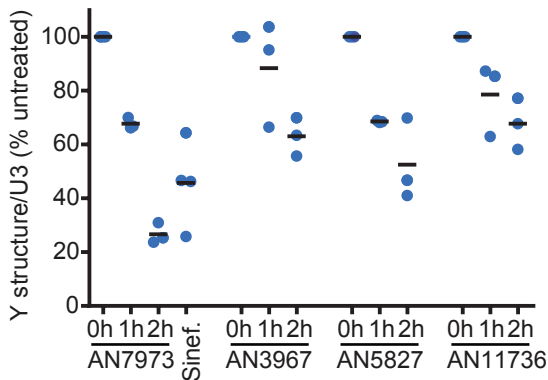

## B. Y structure levels procyclic

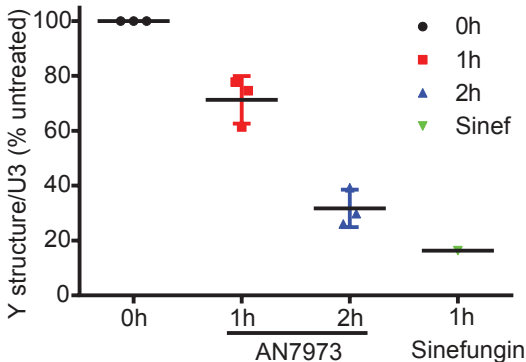

## C. 95% confidence Intervals

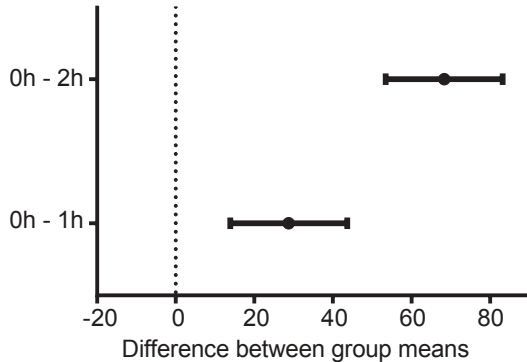

Supplement: S4 Fig — A. Time course of effects of AN7973, AN3967, AN5827 and AN11736 in bloodstream forms (Experiment 2 in Fig 7). Sinefungin treatment was for 30 min. B, C: effect of AN7973 in procyclic forms. (PDF) [file ppat.1007315.s013.pdf]

**A.**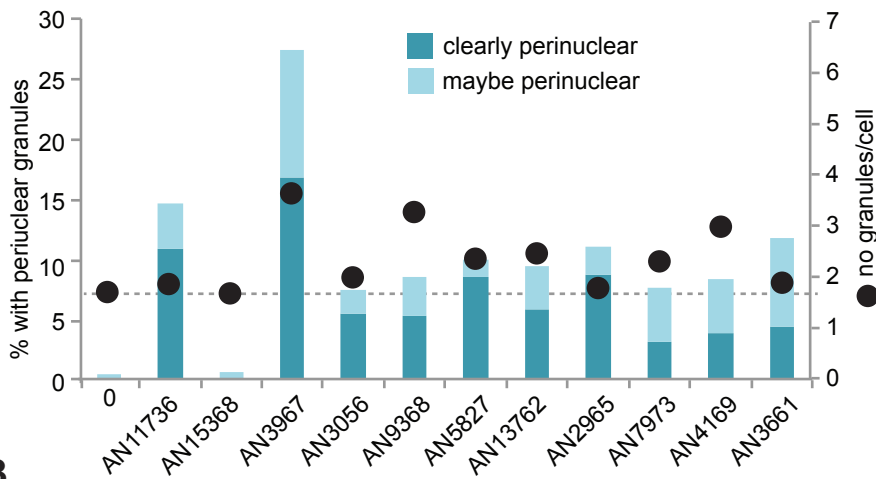**B.**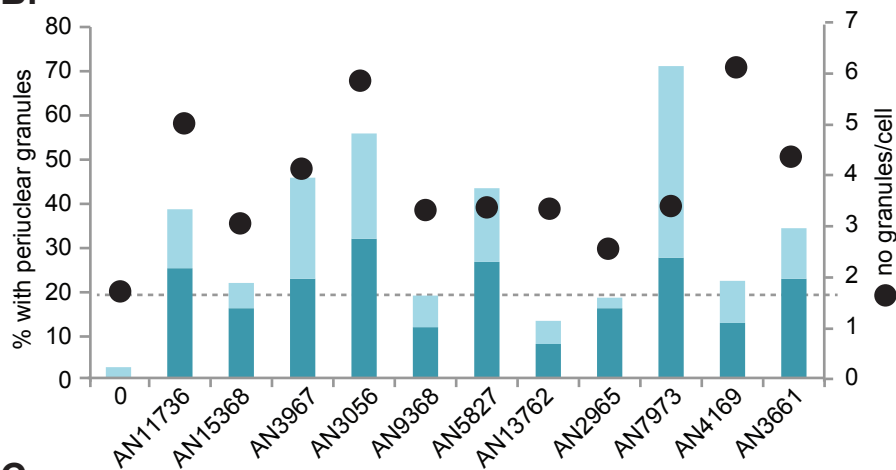**C.**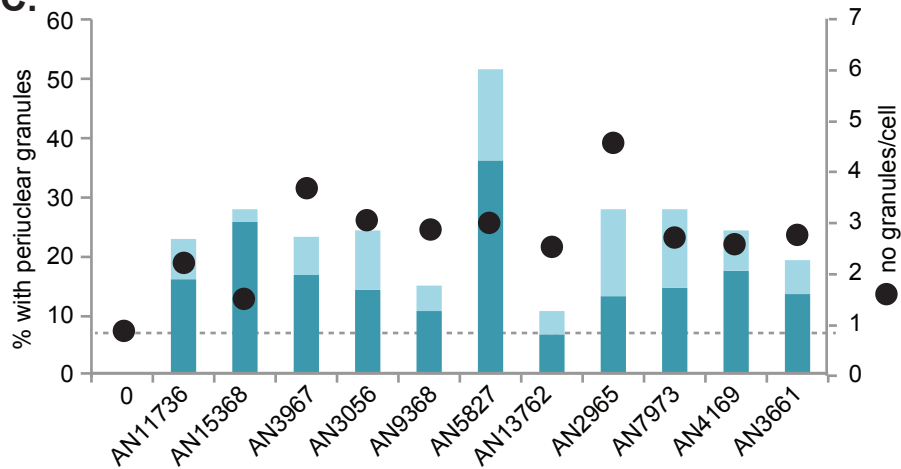

Supplement: S5 Fig — Results for three separate experiments (A, B and C) in which the location of YFP-DHH1 was analysed. The average percentages of cells with clear (dark blue) or possible (pale blue) peri-nuclear granules are plotted as bars, with the average numbers of large granules (at least 4 contiguous pixels at maximum intensity) anywhere in the cell displayed as black spots. The dotted line corresponds to the negative control. (PDF) [file ppat.1007315.s014.pdf]

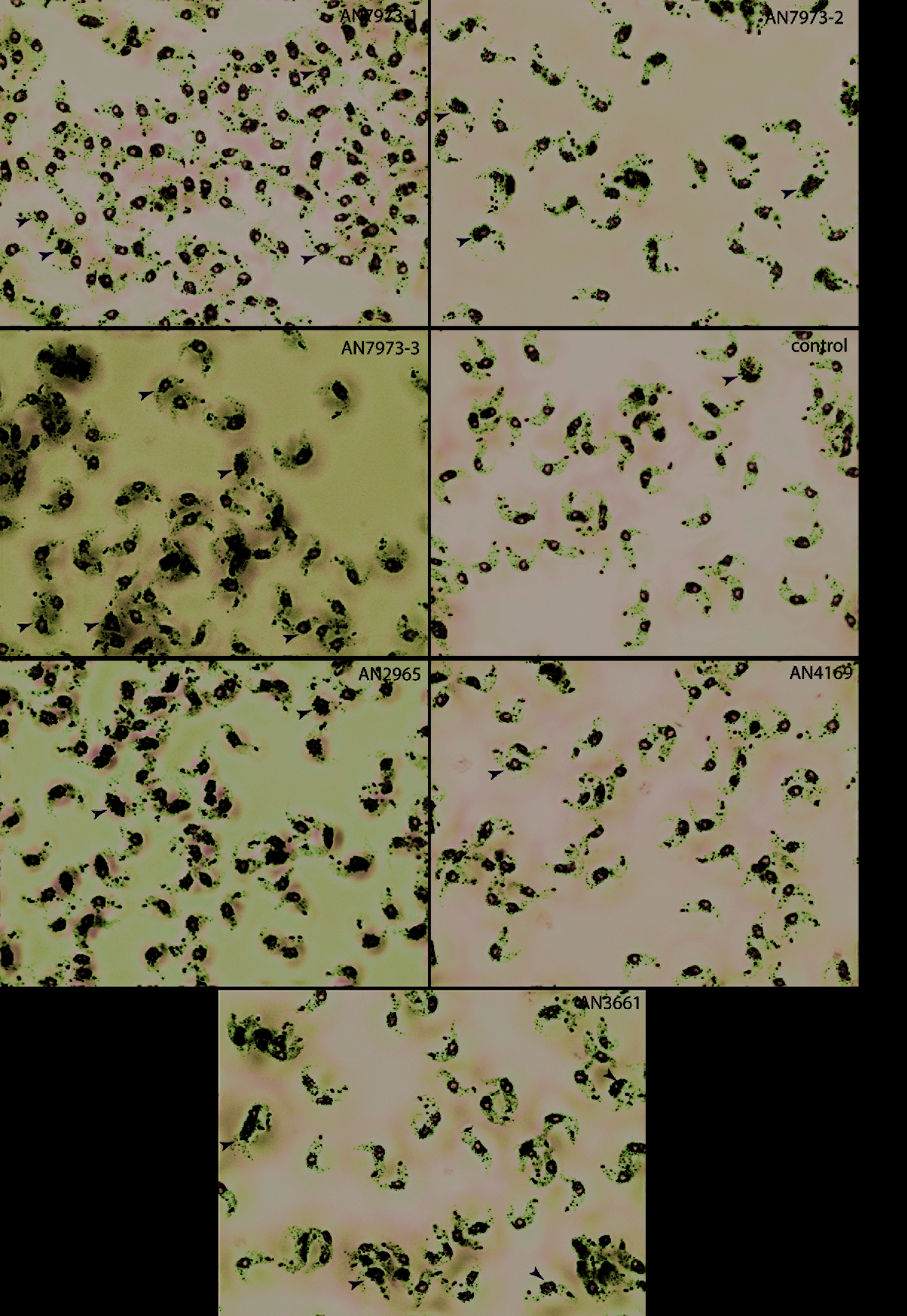

Supplement: S6 Fig — These are not "typical" images; instead, fields in which nuclear periphery granules were present have been chosen. Some (but not all) examples of peri-nuclear granule patterns are indicated by arrows. Compounds used are shown on each image. For AN7973 examples from the three different experiments are shown. The key is on the bottom left. (TIF) [file ppat.1007315.s015.tif]

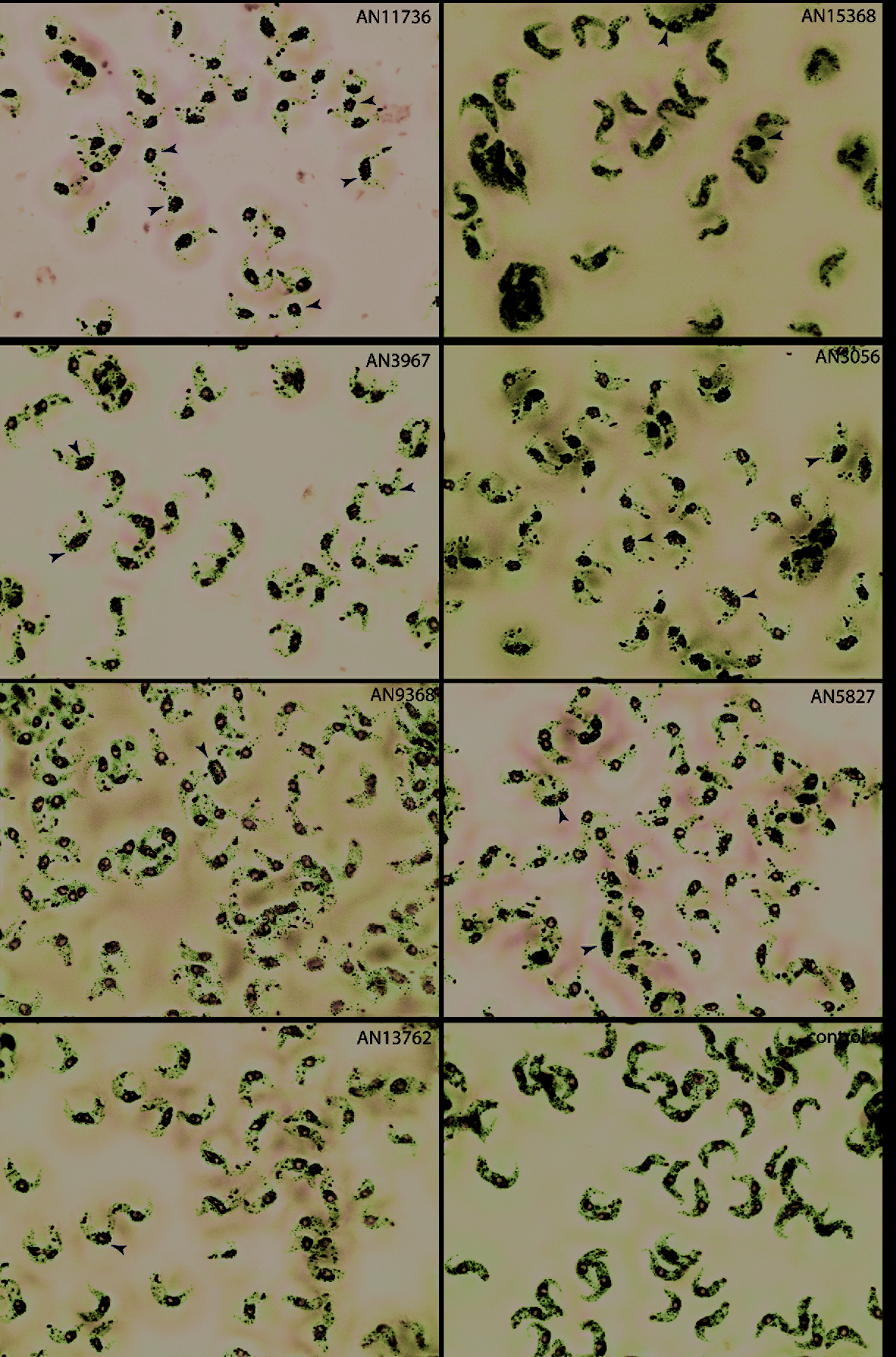

Supplement: S7 Fig — These are not "typical" images; instead, fields in which nuclear periphery granules were present have been chosen. Compounds used are indicated and the key is as in S6 Fig. The control image in this case had brighter YFP fluorescence, for unknown reasons. (TIF) [file ppat.1007315.s016.tif]

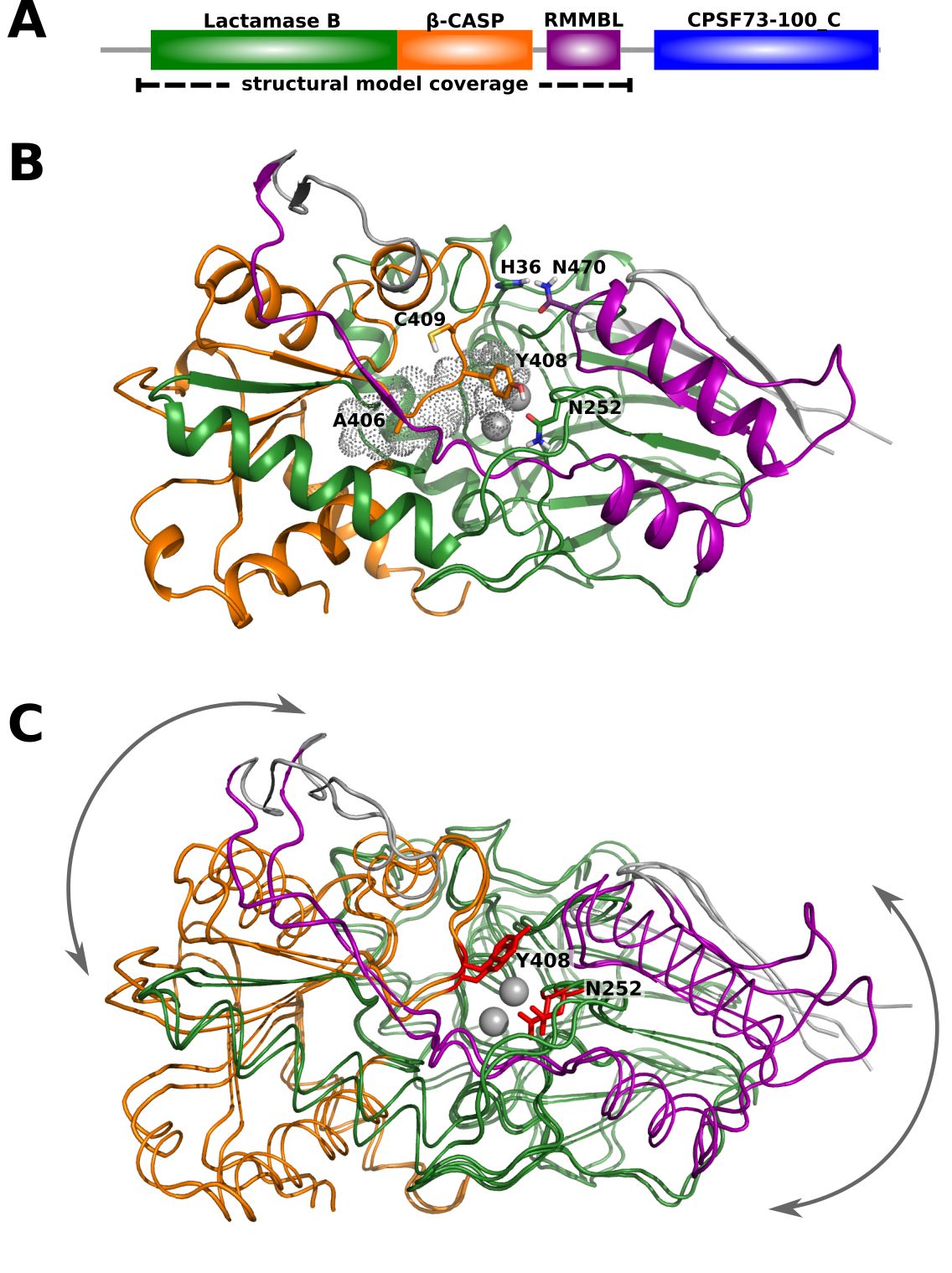

Supplement: S9 Fig — A. Conserved domains found in CPSF3 adapted from the Pfam domain annotations. RMMBL: Zn-dependent metallo-hydrolase RNA specificity domain; CPSF73-100_C: C-terminal conserved region of the pre-mRNA 3'-end-processing of the polyadenylation factor CPSF-73/CPSF-100 proteins. As indicated, this C-terminal domain is not present in the structural model. B. Homology model of TbCPSF3 in cartoon representation colored according to the domain annotations in (A). Residues associated with resistance to benzoxaboroles are highlighted as sticks; the residue numbering corresponds to the P. falciparum sequence. Grey dots indicate the predicted binding pocket for AN7973 for reference. C. Overlay of two snapshots from the first (slowest) mode obtained by normal mode analysis of the TbCPSF3 model structure. The protein is shown in ribbon representation and colored according to the domain annotations in (A). The modes show a general relative rotational breathing motion of the domains as indicated by the arrows. Two important residues lining the interdomain contact region, N252 and Y408 (P. falciparum numbering) are shown as red sticks for reference. (PNG) [file ppat.1007315.s018.png]

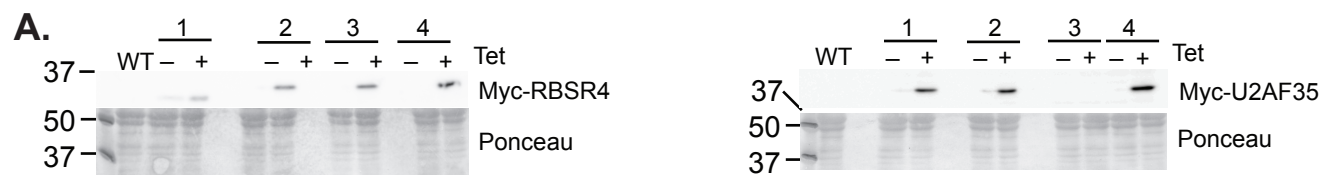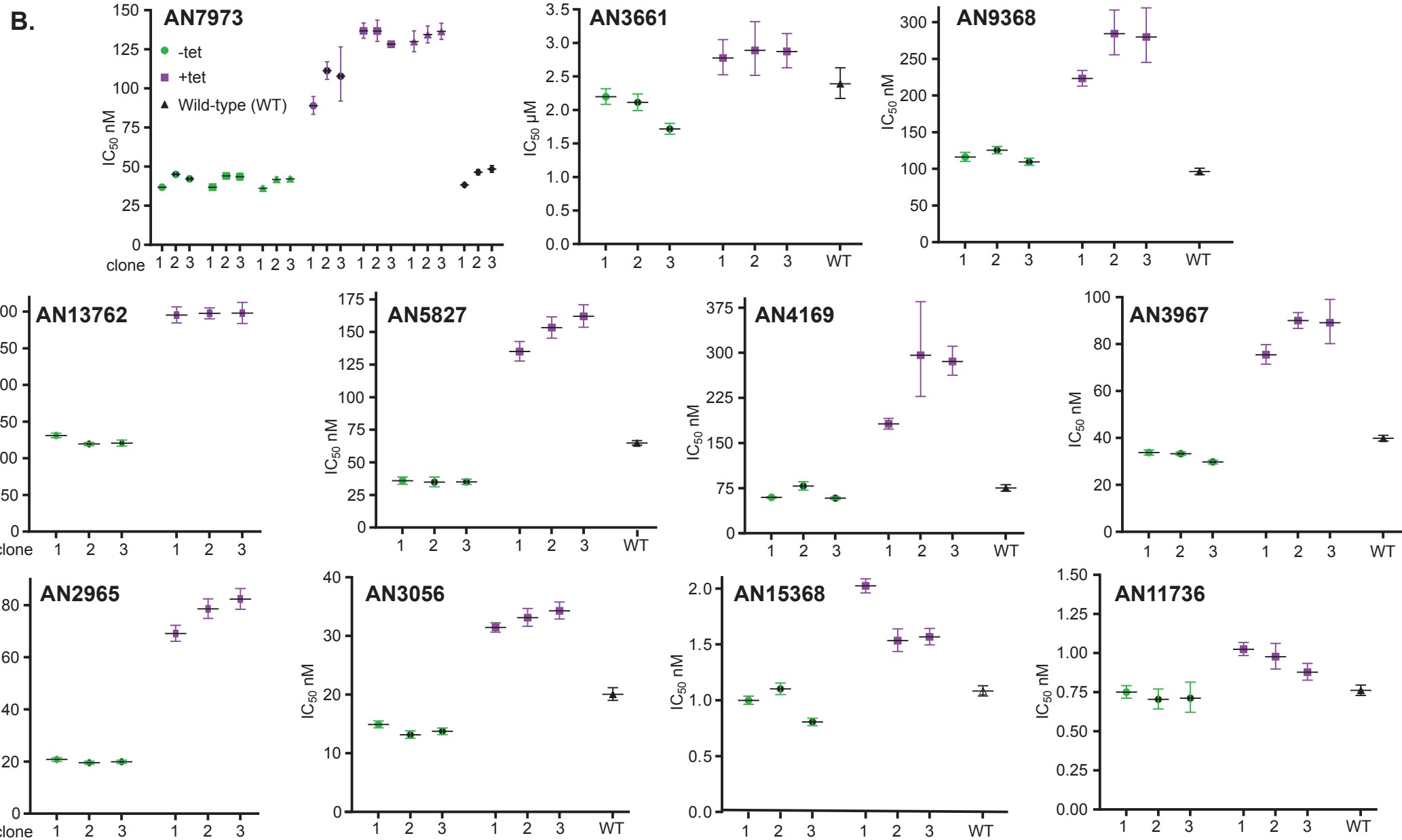

Supplement: S10 Fig — (A) Induced expression of Myc-tagged RBSR4 and U2AF35. (B) IC50 measurements in cells with tetracycline-inducible expression of myc-CPFS3. (PDF) [file ppat.1007315.s019.pdf]

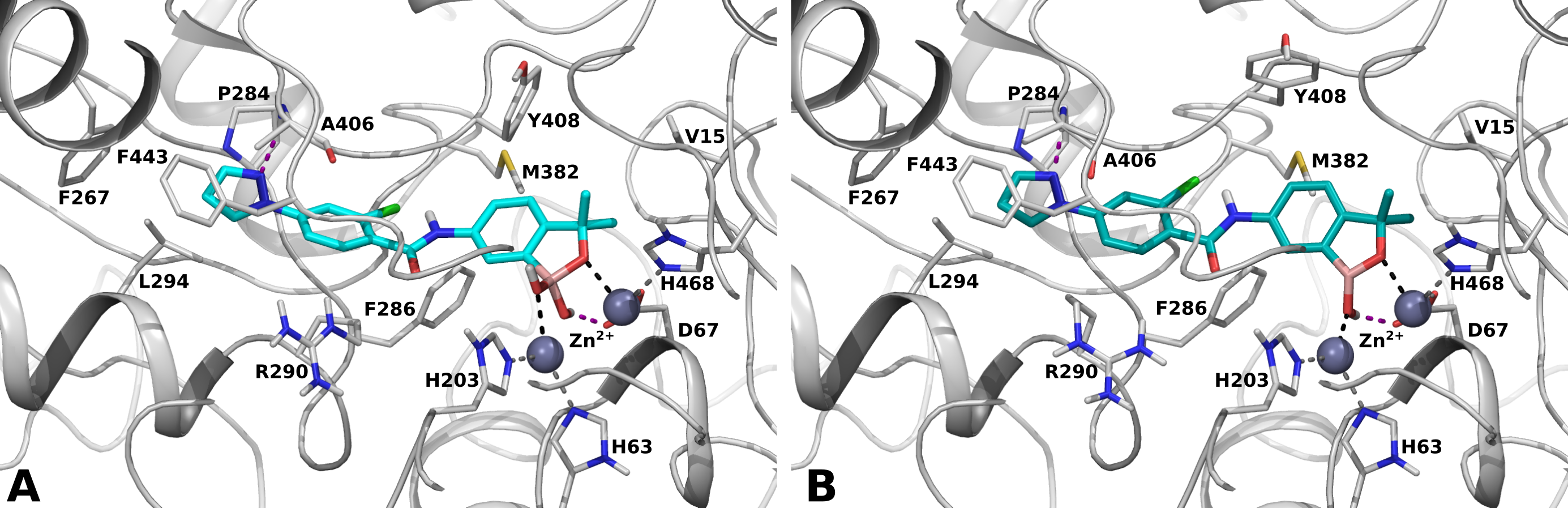

Supplement: S11 Fig — AN7973 (cyan sticks) was docked in the negatively charged form (A, tetrahedral geometry) and the neutral form (B, trigonal planar geometry). In both cases, the top scoring poses with Glide docking scores of -9.3 kcal/mol and -8.4 kcal/mol, respectively, are shown. For the tetrahedral geometry, in total, two poses with an average docking score of -9.0±0.4 kcal/mol and for the planar geometry, three poses with an average docking score of -6.9±1.4 kcal/mol were obtained. The TbCPSF3 homology model is shown in grey cartoon representation with important interacting residues highlighted as sticks and the zinc ions as grey spheres. Dashed lines indicate metal-coordination bonds (grey), metal-ligand interactions (black) and hydrogen bonds to the ligand (purple). The residue numbers correspond to the Plasmodium falciparum CPSF3 sequence. (PNG) [file ppat.1007315.s020.png]

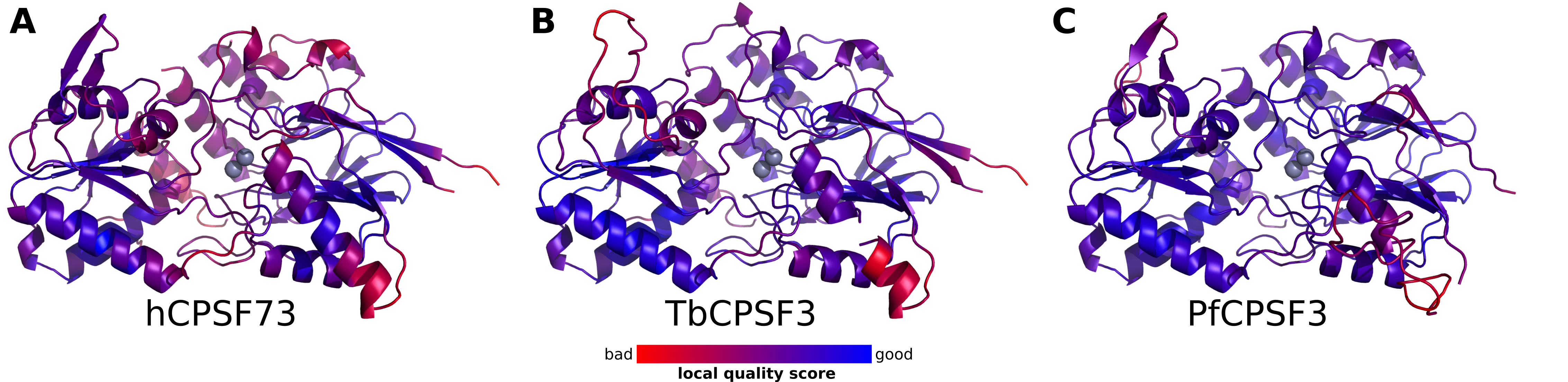

Supplement: S12 Fig — Proteins are shown in cartoon representation. The location of the binding site is indicated by the zinc ions, shown as grey spheres. Local quality estimates are color coded from red (worst) to blue (best). A. human template structure 2i7v, hCPSF73; B. TbCPSF3; C. PfCPSF3. (PNG) [file ppat.1007315.s021.png]
