# Supplementary material for: The trypanocidal benzoxaborole AN7973 inhibits trypanosome mRNA processing
Source: PLoS Pathog. 2018 Sep 25;14(9):e1007315. doi: 10.1371/journal.ppat.1007315 (PMC6173450; doi:10.1371/journal.ppat.1007315)
Supplement: S8 Fig — Only the conserved region is shown. Black-highlighted residues are identical in at least four sequences and blue ones indicate conservative replacements. For key to symbols and numbers see the bottom of the Fig. The gap in the T. vivax sequence is present because of a gap in the genome assembly. (PDF) [file ppat.1007315.s017.pdf]

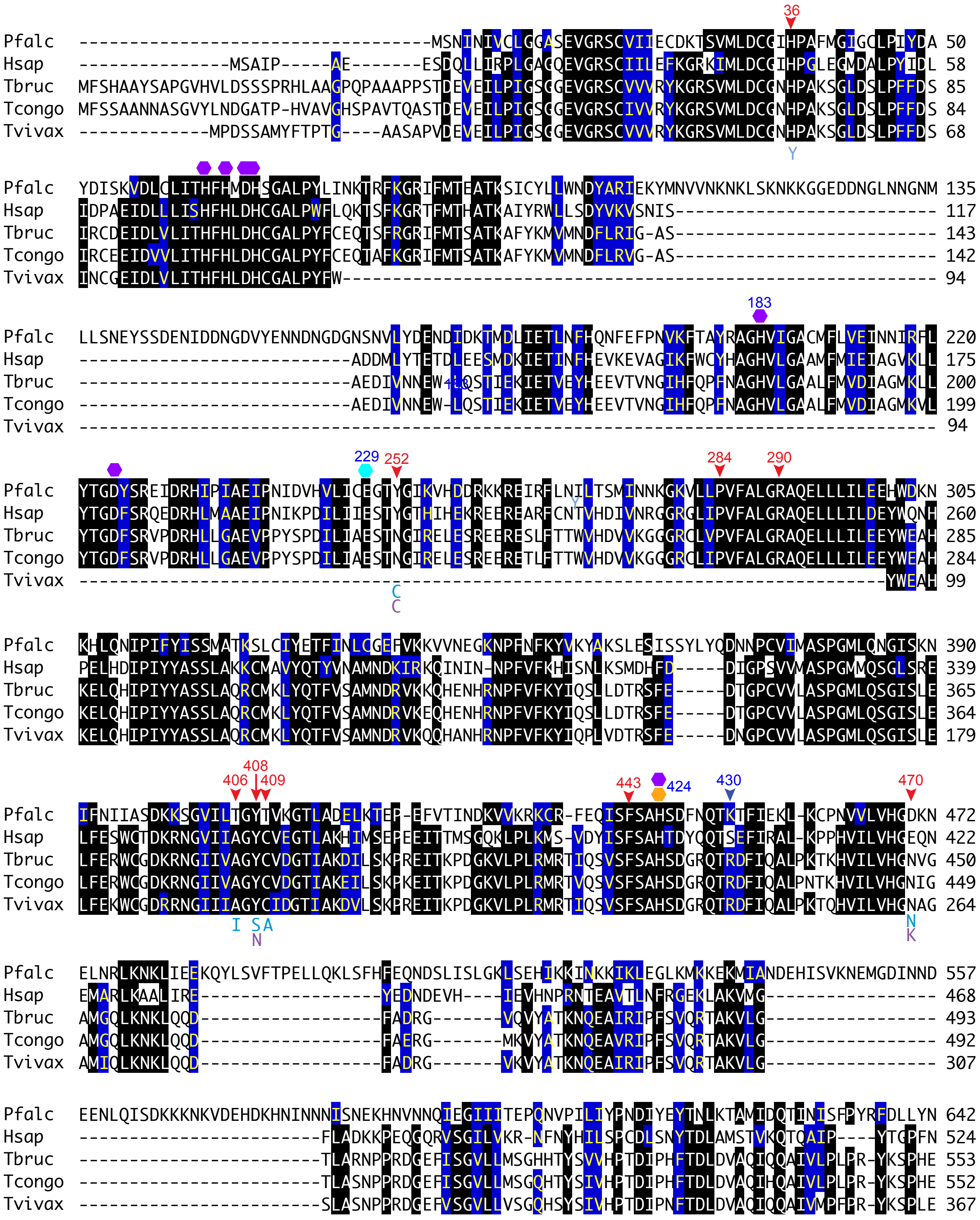

KEY

Conserved chemical group | Identical

Residue change linked to AN3661 resistance in *Toxoplasma*

Residue change linked to AN3661 resistance in *Plasmodium*

Residue number in the *Plasmodium falciparum* sequence / in *T. brucei* sequence

In a structural model, R290 and Y252 interact with the carboxylate of AN3661

Zinc binding

Interaction with His 396 (human) = 424 *T. brucei*

Interaction with phosphate

Pfalc- *Plasmodium falciparum* Hsap- *Homo sapiens*

Tbruc- *Trypanosoma brucei* Tcongo- *Trypanosoma congolense* Tvivax- *Trypanosoma vivax*
